# DUSP6 is a memory retention feedback regulator of ERK signaling for cellular resilience of human pluripotent stem cells in response to dissociation

**DOI:** 10.1038/s41598-023-32567-8

**Published:** 2023-04-07

**Authors:** Dae Hoon Yoo, Young Sam Im, Ji Young Oh, Dayeon Gil, Yong-Ou Kim

**Affiliations:** 1 Division of Intractable Disease Research, Korea National Institute of Health, Osong, Cheongju, 28160 Republic of Korea; 2 Center for National Stem Cell and Regenerative Medicine 202, Osongsaengmyung 2-Ro, Heundeok-Gu, Cheongju, Chungcheongbuk-Do 28160 Republic of Korea

**Keywords:** Cell biology, Stem cells

## Abstract

Cultured human pluripotent stem cells (hPSCs) grow as colonies that require breakdown into small clumps for further propagation. Although cell death mechanism by single-cell dissociation of hPSCs has been well defined, how hPSCs respond to the deadly stimulus and recover the original status remains unclear. Here we show that dissociation of hPSCs immediately activates ERK, which subsequently activates RSK and induces DUSP6, an ERK-specific phosphatase. Although the activation is transient, DUSP6 expression persists days after passaging. DUSP6 depletion using the CRISPR/Cas9 system reveals that DUSP6 suppresses the ERK activity over the long term. Elevated ERK activity by DUSP6 depletion increases both viability of hPSCs after single-cell dissociation and differentiation propensity towards mesoderm and endoderm lineages. These findings provide new insights into how hPSCs respond to dissociation in order to maintain pluripotency.

## Introduction

Response to stimuli is one of the most important characteristics of living organisms. Human pluripotent stem cells (hPSCs) have the potential to differentiate into all cell types of the body, making them a promising candidate for regenerative medicine, cell-based therapeutics, and disease modeling^1,2^. Various culture conditions have been developed to obtain high-quality hPSCs for clinical applications^3–6^. Studies have shown that culture medium can influence the differentiation potential and propensity of pluripotent stem cells^7,8^. Furthermore, passaging methods may affect the hPSC properties since these cells grow in colonies and single-cell dissociation causes cell death^2,9^. However, the dissociation of colonies into small clumps is an inevitable step in hPSCs culture. Mechanical, chemical, and enzymatic methods have been used to dissociate the hPSC colonies. Since chemicals and enzymes are more likely to dissociate colonies into single cells, they can be highly detrimental to hPSCs, which depend on cell–cell and cell–matrix adhesion for survival. Inhibition of the Rho-associated kinase (ROCK) minimizes the dissociation-induced cell death^9^. The mechanism underlying dissociation-induced cell death is well understood^10,11^. While the significance of cell–cell and cell–matrix adhesion in regulating the self-renewal and pluripotency of stem cells^12–14^, the mechanism through which hPSCs survive this deadly stimulus and restore their cellular identity remains poorly understood.

Extracellular signal-regulated kinase (ERK) is an important signaling molecule downstream of the fibroblast growth factor 2 (FGF2) that is required for hPSCs self-renewal^2^. ERK activates genes involved in pluripotency, metabolism, cell cycle, and translation in human embryonic stem cells (hESCs), which are critical for maintaining the undifferentiated state of hPSCs^15^. ERK depletion causes genomic instability, rapid telomere shortening, dysregulated expression of pluripotency genes, G1 cell cycle arrest, and increased apoptosis in mouse embryonic stem cells (mESCs)^16^. On the other hand, the fibroblast growth factor (FGF) activates the ERK signaling pathway, leading to lineage commitment of mESCs^17^. ERK signaling affects several other processes, such as epithelial invasive motility, multilayering, wound healing, and epithelial-mesenchymal transition (EMT) in other cell types^18–21^. These pieces of evidence indicate that ERK signaling must be tightly regulated to maintain the cellular properties of hPSCs. However, the underlying mechanisms have not been fully understood.

To investigate the mechanism regulating hPSCs response to dissociation, we compared the gene expression signature of hESCs and human induced pluripotent stem cells (hiPSCs) that have been passaged using mechanical or chemical dissociation methods. Furthermore, to assess the role of ERK activation and feedback loop, we used the CRISPR/Cas9 system to deplete the dual-specificity phosphatase 6 (DUSP6) in hiPSCs. In this study, we suggest that DUSP6 is a memory-retention feedback regulator of ERK signaling for cellular resilience of hPSCs after cell dissociation.

## Results

### Analysis of differentially expressed genes in cells cultured using different passaging methods

**Figure 1:**
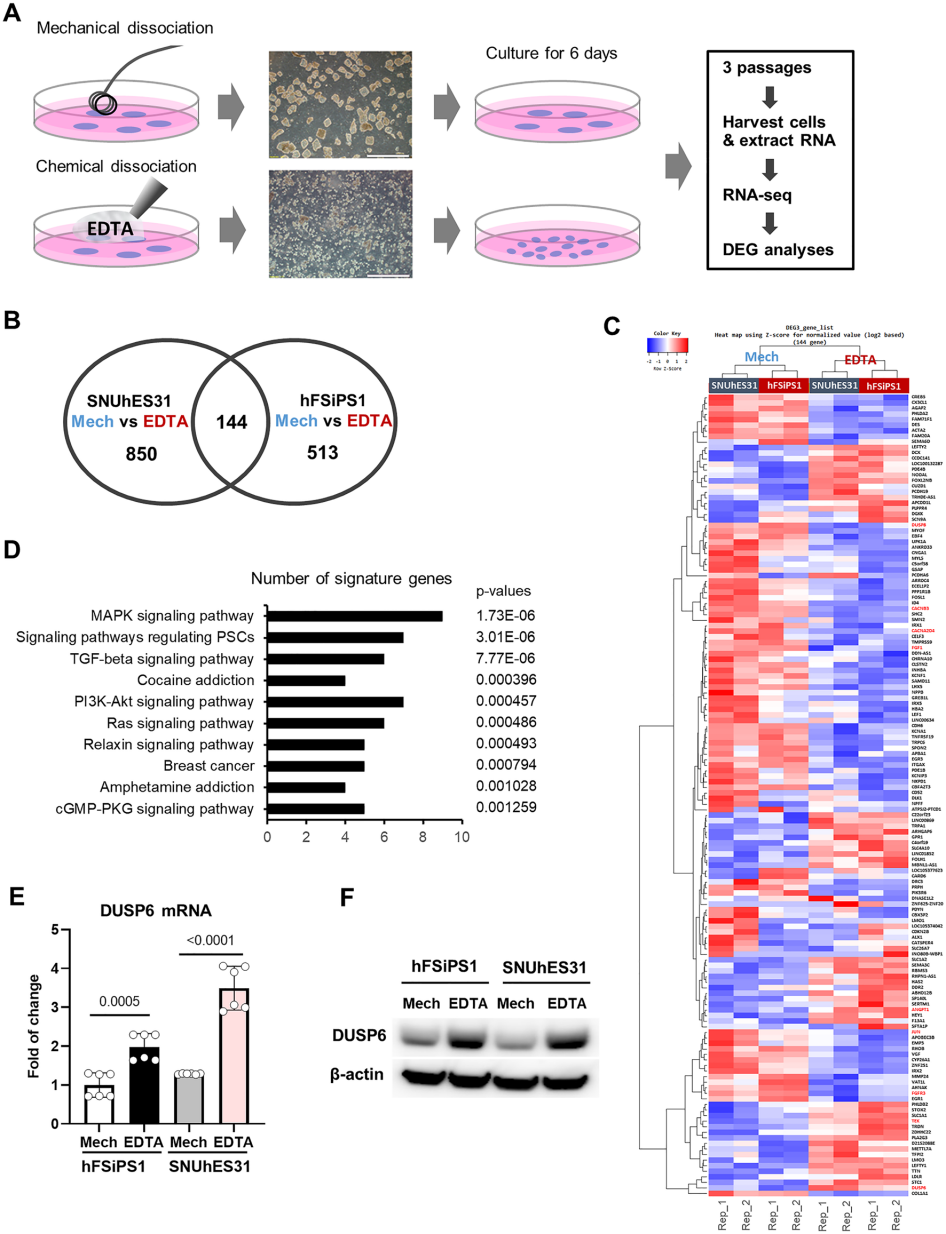
Cell passaging with EDTA increases DUSP6 expression. (**A**) Illustration depicting the experimental setup. hPSCs were passaged using the EZPassage tool for mechanical passaging (Mech) and 0.5 mM EDTA in DPBS for chemical passaging (EDTA). After three passages, hPSCs were harvested and RNA-seq analysis was performed. Clumps obtained after dissociation by each method are shown (scale bar, 1 mm). Samples were used in duplicates. (**B**) Differentially expressed genes (DEGs) in hFSiPS1 and SNUhES31 cells passaged using the mechanical and chemical methods. DEGs were selected on the basis of fold change (> 1.8) and p-values (< 0.05) (**C**) Heatmap of DEGs. Genes associated with MAPK signaling are marked in red. (**D**) Gene Ontology analysis of DEGs revealed that genes regulating the MAPK signaling pathway are significantly enriched. (**E**) Evaluation of *DUSP6* mRNA expression in mechanically and chemically passaged cells (n = 6) by real-time PCR. (**F**) Levels of DUSP6 protein were analyzed by western blotting.

To investigate how hPSCs respond to dissociation, we analyzed the differences in transcriptomes after three passages using mechanical and chemical passaging methods. Two cell lines, hFSiPS1 (hiPSC line) and SNUhES31 (hESC line), were cultured using the mechanical or chemical passaging method (Fig. 1A). Cells were harvested on day 6 after three passages, and gene expression was analyzed by RNA sequencing (RNA-seq). The top 144 differentially expressed genes (DEGs) were selected (fold change > 1.8, p < 0.05) (Fig. 1B,C, DEGs list in Supplemental Table) from both cell lines for further analysis. Pathway analysis of the DEGs showed that genes regulating mitogen-activated protein kinase (MAPK) signaling pathway are highly responsive to cell dissociation (Fig. 1D). We selected DUSP6 to verify the expression because DUSP6 showed the highest expression among all the common 144 DEGs. The expression of DUSP6 was significantly higher in both hFSiPS1 and SNUhES31 cells cultured using the chemical passaging method than cells cultured using the mechanical passaging (Fig. 1E,F).

### Cell dissociation leads to ERK activation in hPSCs

**Figure 2:**
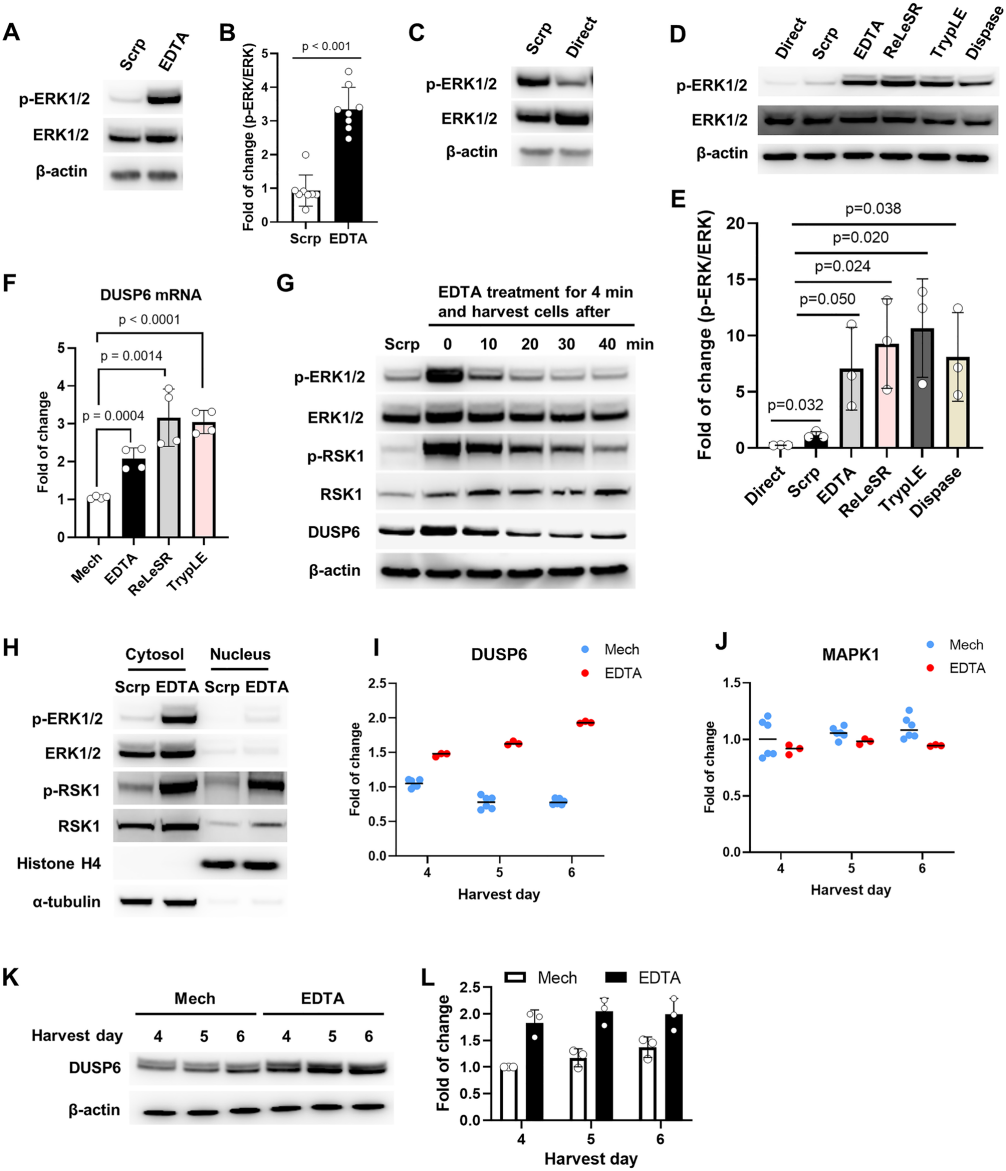
ERK phosphorylation following EDTA-induced cell dissociation in hiPSCs. (**A**) hFSiPS1 cells were harvested using a scraper (Scrp) or EDTA and phosphorylated ERK (p-ERK) levels were analyzed. Cells were treated with EDTA for 4 min before harvesting. (**B**) p-ERK to ERK ratio was calculated (n = 8). (**C**) p-ERK levels in cells harvested using a scrapper (Scrp) and cells that were directly lysed (Direct) on culture dishes. For direct lysis, cells were washed with DPBS twice and lysed using the lysis buffer containing phosphatase inhibitors. (**D**) p-ERK levels in cells harvested by Direct, Scrp, EDTA, ReLeSR, TrypLE and Dispase. (**E**) Quantification of p-ERK levels (n = 3). (**F**) Expression of *DUSP6* mRNA was analyzed using real-time PCR after cells were passaged thrice using the mechanical method, EDTA method, ReLeSR, or TrypLE. (**G**) Time course showing the levels of p-ERK, phosphorylated RSK1 (p-RSK1), and DUSP6 following 4 min of EDTA treatment. After the treatment, EDTA solution was removed and cells were washed with DPBS. Cells remained in DPBS until harvest at indicated time points. (H) Analysis of nuclear translocation of p-ERK and p-RSK1 following EDTA treatment. Cells were harvested after 4 min of EDTA treatment and washed with DPBS. Nuclear and cytosolic fractions were isolated. Histone H4 was used as the nucleus indicator; α-tubulin was used as the cytosol indicator. (I) Cells were harvested on indicated day after mechanical or chemical passaging. *DUSP6* expression was analyzed using real-time PCR. (J) *MAPK1* was used as a control for both passaging methods. (K) DUSP6 protein levels in cells harvested on indicated day after mechanical or chemical passaging. (I) Quantification of DUSP6 protein (n = 3).

Since DUSP6 expression is regulated via ERK signaling downstream of the fibroblast growth factor receptor (FGFR)^23^, we examined the effect of cell dissociation on ERK. Cells were harvested using a scraper or EDTA, and phosphorylated ERK (p-ERK) levels were analyzed by western blotting. Results showed that in cells harvested using EDTA, p-ERK levels were more than threefold higher compared to cells harvested using the scraper (Fig. 2A,B). Interestingly, cells harvested using the scraper had higher levels of p-ERK compared to cells that were directly lysed on the culture dish (Fig. 2C). To confirm whether other widely used cell-dissociation reagents also activate ERK, we analyzed p-ERK levels of harvested cells using ReLeSR, TrypLE, and Dispase. Results showed that these reagents also activate ERK, indicating that passaging methods involving dissociation are accompanied by ERK activation (Fig. 2D,E). Furthermore, qRT-PCR analysis confirmed that cells harvested on day 5 after four passages using ReLeSR and TrypLE had increased *DUSP6* mRNA expression levels compared to mechanically passaged cells (Fig. 2F). To evaluate the duration time of the ERK activation by dissociation, we analyzed level of p-ERK after EDTA treatment. ERK activation following EDTA treatment was immediate and lasted less than 10 min (Fig. 2G). DUSP6 protein levels were decreased when p-ERK levels were reduced. The ribosomal S6 kinase 1 (RSK1), a downstream target of ERK^24^, was also activated following cell dissociation; however, phosphorylated RSK1 (p-RSK1) persisted for slightly longer time than p-ERK (Fig. 2G). To identify the nuclear signaling molecules, we analyzed the sub-cellular localization of p-ERK and p-RSK1. As shown in Fig. 2H, p-RSK1 was localized in the cytosol and nucleus of EDTA-treated cells, whereas p-ERK was mostly localized in the cytosol, indicating that RSK1 might be the main nuclear signal transducer for dissociation-induced ERK signaling. In the respect that ERK-activated RSK is necessary to induce mesenchymal motility and invasive capacities in epithelial and carcinoma cells^24^, the activated RSK might provide an advantage in survival of hPSCs during dissociation. Next, we investigated how transient ERK activation in cells passaged using EDTA led to an increase in DUSP6 expression even until day 6 after passaging. To address this, cells were harvested on day 4–6 after three rounds of mechanical or EDTA passaging and the expression of *DUSP6* mRNA was analyzed. As shown in Fig. 2I, *DUSP6* expression was continuously increased until day 6 in cells passaged using EDTA, whereas the expression was slightly reduced in cells passaged using the mechanical method. There was no change in the expression of *MAPK1*, which was used as a negative control for both passaging methods (Fig. 2J). Western blot analysis confirmed that DUSP6 protein were maintained in higher level in EDTA-passaged cells during the post-passage period (Fig. 2K,L). These results indicate that cell dissociation transiently activates ERK, however, long-term DUSP6 expression is not directly regulated by the transient activation. Expression of DUSP6 promoter is known to be regulated by E twenty-six (ETS) factors which are also known targets of ERK signaling^25^. However, whether long-term expression of DUSP6 is regulated by ETS factors needs to be clarified. Long-term expression of DUSP6 seems important to keep ERK activity low because phosphatase activity of DUSP6 is inactivated during dephosphorylation of ERK^26^.

### Upstream signals regulating ERK activation following cell dissociation

**Figure 3:**
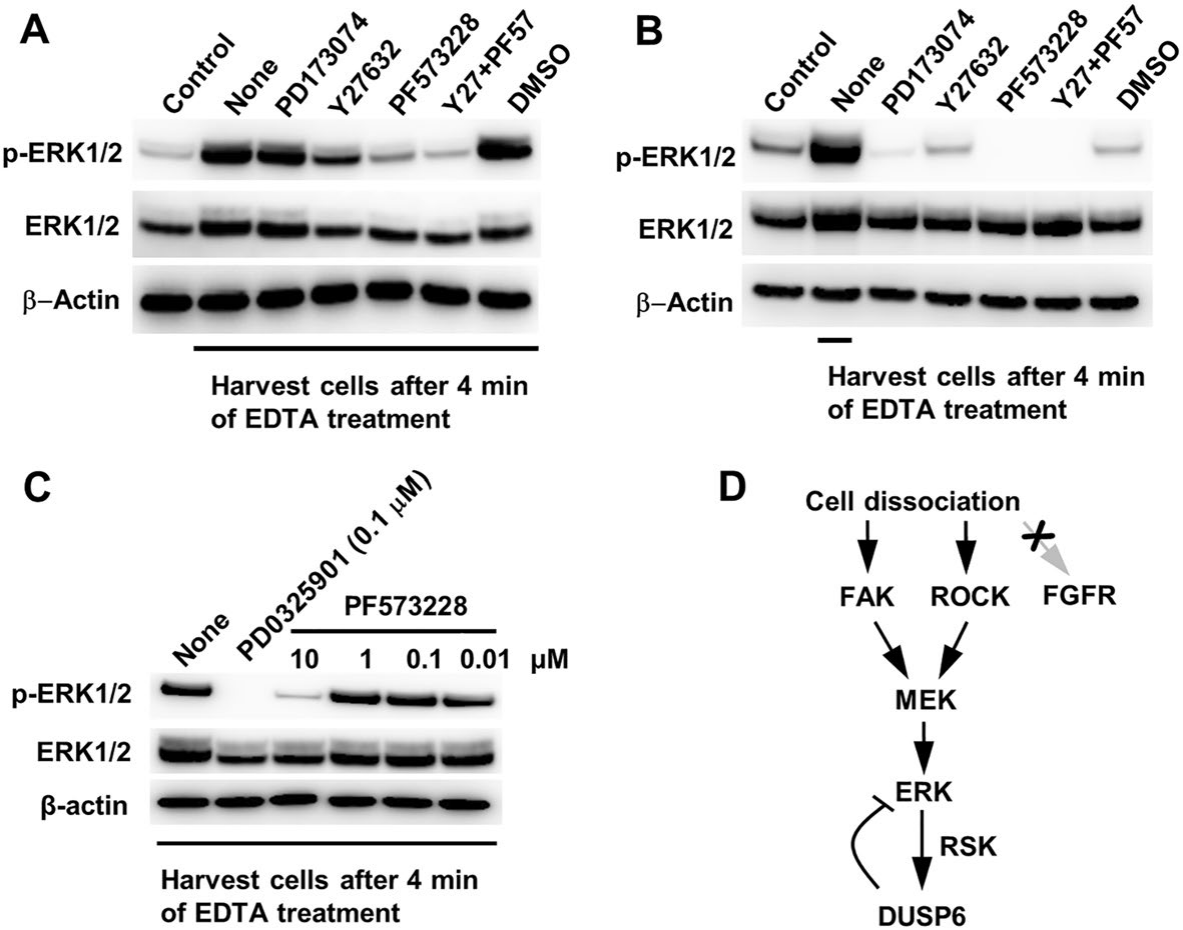
Identification of upstream signals regulating ERK activation in dissociated cells. (**A**) Cells were treated with indicated inhibitors for 1 h before EDTA treatment. Cells were harvested and western blotting was performed. “Control” indicates cells harvested without EDTA treatment. “None” indicates cells harvested after EDTA treatment without treatment of inhibitor. Cells were harvested using scrapper. Following inhibitors were used: PD173074 (0.1 μM) for FGFR, Y27632 (10 μM) for ROCK, and PF573228 (10 μM) for FAK. DMSO was used as a mock (**B**) Cells were treated with indicated inhibitors for 1 h and harvested without EDTA treatment. “None” indicates cells harvested after EDTA treatment without treatment of inhibitor. (**C**) Estimation of optimal PF573228 concentration for ERK suppression following EDTA treatment. Cells were treated with indicated concentrations of PF573228 for 1 h before EDTA treatment. (**D**) Possible signaling pathway regulating ERK activation following cell–cell and cell–matrix dissociation.

Since dissociation of hPSC colonies is known to activate ROCK^9^, we analyzed whether dissociation-induced ERK activation can be suppressed using inhibitors. PF573228, an inhibitor of the focal adhesion kinase (FAK), clearly suppressed the dissociation-induced ERK activation, whereas the ROCK inhibitor, Y27632, slightly suppressed the ERK activation (Fig. 3A,B). Meanwhile, the FGFR inhibitor, PD173074, was not able to suppress the dissociation-induced ERK activation although it was able to suppress non-dissociation-related ERK activity when added without dissociation, indicating that unlike FGF signaling through FGFR, dissociation-induced ERK activation does not come through FGFR. Simultaneous treatment with the ROCK and FAK inhibitors showed a synergistic effect on ERK activation, indicating that the ERK activation signal following cell dissociation may originate from at least two independent pathways: one from the disrupted cell-ECM adhesion through FAK and the other from dissociation-activated ROCK (or indirectly from ROCK-induced blebbing). Since high concentration of PF573228 is known to partially inhibit other kinases, including ERK and MEK^27^, we tested various concentrations of this inhibitor for the suppression effect (Fig. 3C). PF573228 prevented dissociation-induced ERK activation only at 10 μM concentration. Therefore, the involvement of FAK in dissociation-induced ERK activation seems to need further verification. However, because FAK is localized between membrane integrins and the actin cytoskeleton, and membrane detachment from the actin cytoskeleton leads to the formation of blebs^28^, the involvement of FAK as a sensor or signal transducer during cell detachment is highly plausible. The model of the signaling pathway regulating dissociation-induced ERK activation and its feedback regulation by DUSP6 is depicted in Fig. 3D.

### DUSP6 is a memory retention feedback regulator of basal ERK signaling

**Figure 4:**
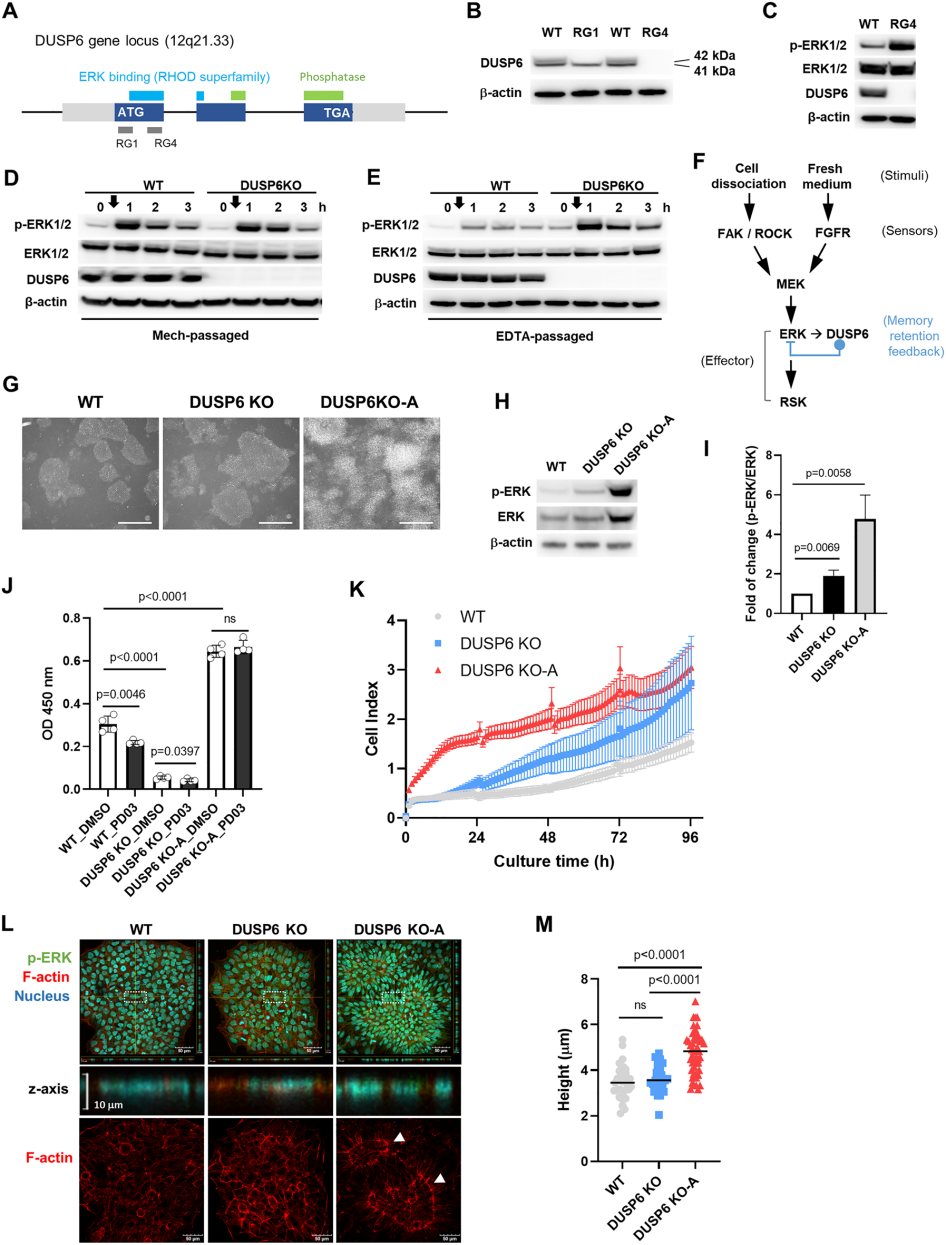
Generation and characterization of *DUSP6* knockout cells. (**A**) Target sites of RG1 and RG4 sgRNAs in the first exon of the *DUSP6* gene locus. Target sites were selected using the in vitro T7E1 assay. See Supplemental Fig. S1. (**B**) Western blot analysis using the anti-DUSP6 antibody. Knockout cells generated using the RG1 sgRNA express a smaller version of DUSP6 protein, while those generated using the RG4 sgRNA are depleted of DUSP6 protein. Estimated molecular weight of each band is indicated. (**C**) Levels of p-ERK, ERK, and DUSP6 in WT and *DUSP6* KO cells generated using the RG4 sgRNA. (**D**) Western blot analysis for ERK activity in WT and *DUSP6* KO cells which are passaged mechanically (Mech-passaged). Arrows indicate the time points of replacing with fresh medium. (**E**) Western blot analysis for ERK activity in WT and *DUSP6* KO cells which are passaged by EDTA (EDTA-passaged). Arrows indicate the time points of replacing with fresh medium. (**F**) Schematic diagram representing ERK signaling regulation following cell dissociation and addition of fresh medium. DUSP6 feedback regulation is depicted as memory retention feedback because DUSP6 regulates the basal ERK activity over the long term following EDTA passaging, which induces DUSP6 expression. (**G**) Morphology of colonies formed by the *DUSP6* KO subline (KO-A) (scale bar, 500 μm). (**H**) ERK activity in the two *DUSP6* KO sublines. A representative western blot result is shown. (**I**) Bar graph depicting p-ERK/ERK ratio (n = 3). (**J**) Comparison of survival rates between WT and *DUSP6* KO cells after EDTA passaging and effect of MEK inhibition. EDTA-passaged cells (on day 6 after passaging) were treated either with DMSO or PD0325901 for 1 h before EDTA treatment for additional passaging. After EDTA treatment and washing, 5 × 10^4^ cells per well were seeded into 96 well plate. After 24 h, survived cells were estimated using CCK-8 assay (n = 8). (**K**) Growth curves of WT and *DUSP6* KO cells. Growth curves were examined using real-time cell analysis (RTCA) system (n = 4). Cells were seeded on the E-plate 96. Cell Index is a dimensionless parameter derived as a relative change in measured electrical impedance. (**L**) Confocal images of WT and *DUSP6* KO cells. Colonies were stained for p-ERK (green), filamentous actin (red), and nucleus (blue). Images were acquired through the z-axis and merged. Filamentous actin (F-actin) ring structures between the center and periphery of the colony are indicated arrows. (**M**) Height of the WT and *DUSP6* knockout cell colonies. P-values are indicated on the graph. Ns: not significant.

To elucidate the role of ERK activation and long-term expression of DUSP6 in cells passaged using EDTA, we established *DUSP6* knockout (KO) cell lines from hFSiPS1 using the CRISPR/Cas9 system. We designed single guide RNAs (sgRNAs) for targeting four different sites in the first exon of the *DUSP6* gene (Supplemental Fig. S1). After validation, we targeted two sites within the first exon of *DUSP6* with RG1 and RG4 sgRNAs (Fig. 4A). Deletion of the targeted sequences was confirmed by Sanger sequencing (Supplemental Fig. S2A). Depletion of the DUSP6 protein was confirmed by western blotting (Fig. 4B). Western blot analysis showed two bands of DUSP6 protein with similar molecular weight. Only upper band disappeared in *DUSP6* KO cells generated using RG1 while both bands disappeared in *DUSP6* KO cells generated using RG4, indicating that the protein of lower band might be translated using the second ATG as the starting codon located at position 40–42 (Supplemental Fig. S2B,C). This result is consistent with the observation in COS-1 cells, where the *DUSP6* transcript uses both the first and second ATG as initiation codons^22^. Therefore, we used *DUSP6* KO cells generated using the RG4 sgRNA for further analysis. Loss of DUSP6 did not affect the expression of pluripotency markers (Supplemental Fig. S3A). However, *DUSP6* KO cells showed higher levels of p-ERK than WT cells (Fig. 4C). Interestingly, the duration of ERK activation following EDTA treatment was not affected (Supplemental Fig. S3B). These results indicate that, following cell dissociation, DUSP6 regulates the basal ERK activity and not the duration of ERK activation. These findings about the role of DUSP6 for the basal ERK activity are consistent with a previous study based on *Dusp6* null mice^29^.

Since cell dissociation and FGFR both seem to share the ERK signaling pathway, we assessed whether cell dissociation affects the FGFR-ERK signaling axis by adding fresh medium containing FGF2. Cells were passaged either mechanically or with EDTA, and were used to evaluate the levels of ERK activation after replacement with fresh media on post-passaging day 6. Cells were then harvested at the indicated time after changing with fresh media. Results showed that p-ERK levels were lower in WT cells compared to the *DUSP6* KO cells passaged using EDTA, whereas no significant difference was observed between WT and *DUSP6* KO cells passaged using the mechanical method (Fig. 4D,E). These results indicate that cells having experienced dissociation by EDTA passaging suppress ERK activity through the long-term expression of DUSP6. Therefore, long-term expression of DUSP6 can be considered as a kind of cellular memory for proper regulation of ERK signaling (Fig. 4F).

During subculture using mechanical or EDTA passaging, mechanically passaged *DUSP6* KO cells showed transformed cell-like outgrowths (Fig. 4G). These cells were termed as the subline *DUSP6* KO-A. Chemical passaging of *DUSP6* KO-A did not reverse the phenotype. Suppression of ERK signaling using a MEK inhibitor, PD0325901, suppressed the outgrowth phenotype, indicating that the phenotype was induced by elevated ERK signaling (Supplemental Fig. S3C). Western blot analysis showed that *DUSP6* KO-A cells have approximately fivefold higher p-ERK levels than WT and twofold higher p-ERK levels than KO cells (Fig. 4H,I). The survival rate of *DUSP6* KO-A cells was approximately twofold higher than that of WT, whereas *DUSP6* KO cells showed lower survival rate than WT cells (Fig. 4J). MEK inhibition using PD0325901 definitely decreased the survival rate of WT and DUSP6 KO cells while there was no effect on DUSP6 KO-A cells. The growth rate of *DUSP6* KO-A cells was also higher than that of WT and *DUSP6* KO cells, especially in the early stages (Fig. 4K). The overall phenotype of *DUSP6* KO-A cells was similar to that of other transformed cells with high ERK activity expressing constitutively active MEK or MEK-ERK2^30,31^. These results indicate that ERK activation following cell dissociation is a pro-survival signal and has a positive role in cell cycle progression, as observed in other cell types^19,32^. Fluorescence confocal microscopy showed high-density cells with increased height in the center region in *DUSP6* KO-A colonies (Fig. 4L,M). Furthermore, *DUSP6* KO-A colonies showed filamentous actin (F-actin) ring structures between the center and periphery of the colony (arrows in Fig. 4L). However, the relationship between ERK activity and the F-actin ring formation remains to be elucidated.

### Elevated ERK activity causes tilting of differentiation propensity

**Figure 5:**
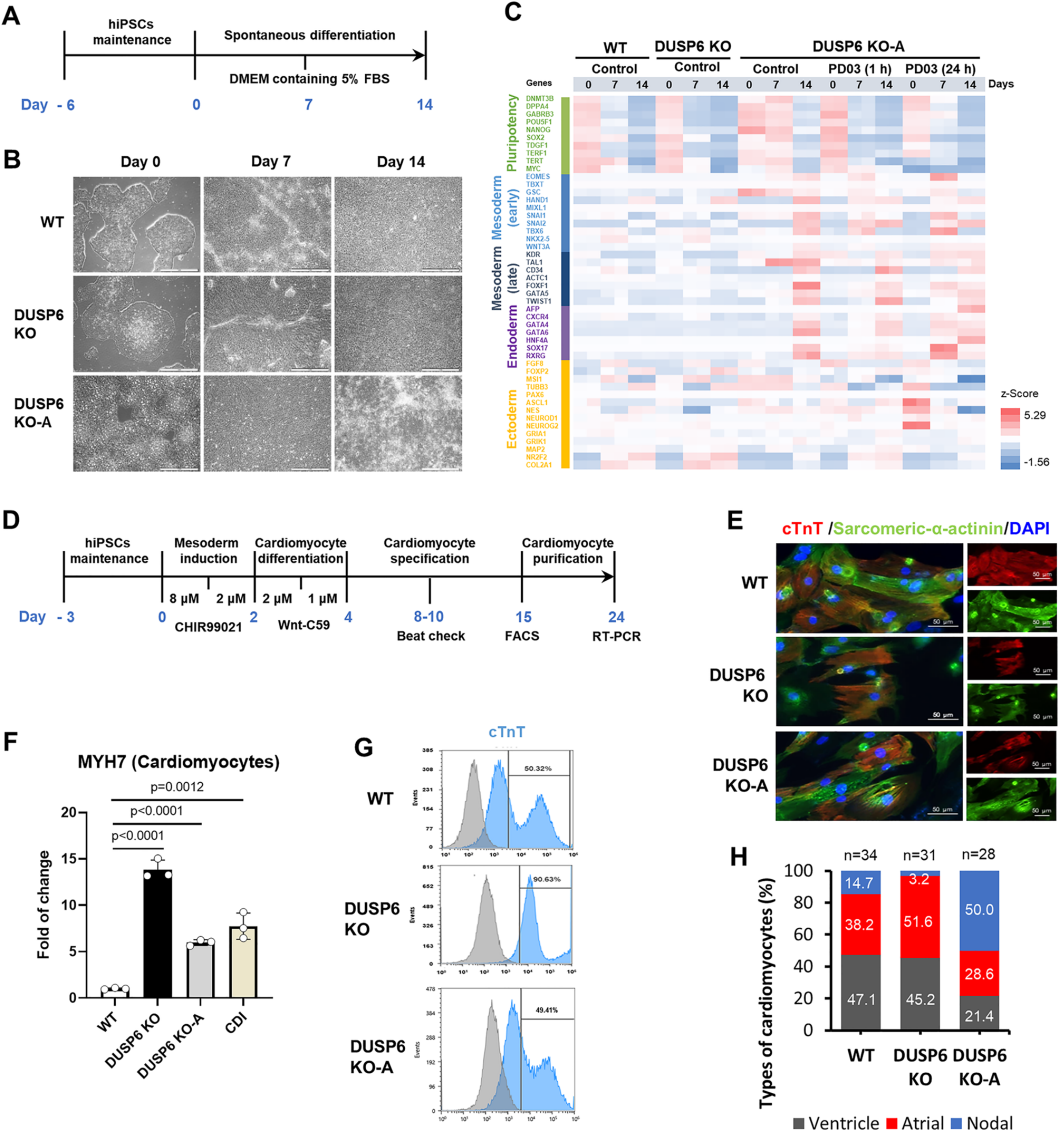
Effect of DUSP6 depletion on hPSC differentiation propensity. (**A**) Spontaneous differentiation scheme. Briefly, cells were maintained in the TeSR-E8 medium and spontaneous differentiation was induced by adding DMEM containing 5% FBS. (**B**) Images of colonies and differentiated cells before harvesting. (**C**) Estimation of differentiation propensities of WT and *DUSP6* KO cells. Heatmap was created with z-Score calculated from RPKM values of RNA-seq data. To suppress the ERK signaling, WT cells were treated with the MEK inhibitor (PD0325901, 1 mM) for 1 h before initiating differentiation. (**D**) Cardiomyocyte differentiation scheme. Briefly, cells were maintained in the StemMACS iPS-BREW XF media. Mesoderm differentiation was induced with CHIR99021 and cardiomyocyte differentiation was induced with Wnt-C59. Cardiomyocyte specification was confirmed by evaluating the cell beating. (**E**) Cardiomyocytes were stained for cardiac troponin T (cTNT), sarcomeric-α-actinin, and DAPI (scale bar, 50 μm). (**F**) Expression of the mature cardiomyocyte marker, MYH7, was measured using real-time PCR (n = 3). mRNAs from cardiomyocytes generated from CDI and primary heart tissue were used a positive control. P-values are indicated. (**G**) Cardiomyocyte differentiation was assessed by evaluating cTnT-positive cells using FACS. (**H**) Types of cardiomyocytes derived from WT, KO, and KO-A cells.

Although ERK activity has been shown to affect the self-renewal and differentiation of mESCs^33,34^, its role in hPSCs remains elusive. In mice, ERK signaling plays an important role in lineage determination during early development^35–37^. To investigate whether enhanced ERK activity in DUSP6-depleted cells affects their differentiation propensity, we induced WT and *DUSP6* KO cells using the spontaneous differentiation method (Fig. 5A,B)^8^. To validate the method, we analyzed the differentiation propensity after treating WT cells with reagents inducing three germ layers: activin A for endoderm, CHIR99021 for endo- and mesoderm and SB431542/dorsomorphin for ectoderm^38–41^. The reagents successfully altered the differentiation propensity of WT cells into endoderm, or endo- and mesoderm, or ectoderm (Fig. S4), indicating that the spontaneous differentiation method can evaluate the differentiation propensity of hPSCs. *DUSP6* KO-A cells showed a significantly increased differentiation propensity towards mesoderm and endoderm lineages (Fig. 5C). The propensity of *DUSP6* KO-A cells remained unchanged even when differentiation was initiated after 1 h or 24 h treatment with the MEK inhibitor to suppress ERK activity. Notably, the suppression of ERK signaling before spontaneous differentiation induced earlier termination of expression of pluripotency marker genes and earlier initiation of expression of mesodermal lineage genes. These results indicate that long-term expression of DUSP6 for the suppression of ERK activity plays an important role in restoring the balanced differentiation propensity. In the teratoma assay, *DUSP6* KO and KO-A cells formed teratomas consisting of all three germ layers (Supplemental Fig. S5). The teratomas formed by *DUSP6* KO-A cells were more than twofold larger than those formed by WT and *DUSP6* KO cells.

Next, we assessed the mesodermal differentiation potential of *DUSP6* KO cells (Fig. 5D). Cardiomyocyte differentiation was confirmed by immunostaining and the presence of beating cells (Fig. 5E and Supplemental Fig. S6A). The expression of the mature cardiomyocyte marker, myosin heavy chain 7 (MYH7), was higher in cardiomyocytes derived from *DUSP6* KO and KO-A cells compared to those derived from WT cells (Fig. 5F). More than 90% cardiomyocytes derived from *DUSP6* KO cells were positive for cardiac troponin T (cTnT), a cardiomyocyte marker, whereas approximately 50% of cardiomyocytes derived from WT and *DUSP6* KO-A cells were cTNT-positive (Fig. 5G). Grouping cardiomyocytes by measuring the beating pattern showed that the percentage of cells with nodal characteristics was lower in cardiomyocyte derived from *DUSP6* KO cells while it was higher in those derived from *DUSP6* KO-A cells (approximately 50%) (Fig. 5H and Supplemental Fig. S6B). These results show that the differentiation ratios of both *DUSP6* KO and KO-A cells into cardiomyocyte are higher than those of WT cells while their characteristics are somewhat different.

### DNA methylation changes in KO-A cells

**Figure 6:**
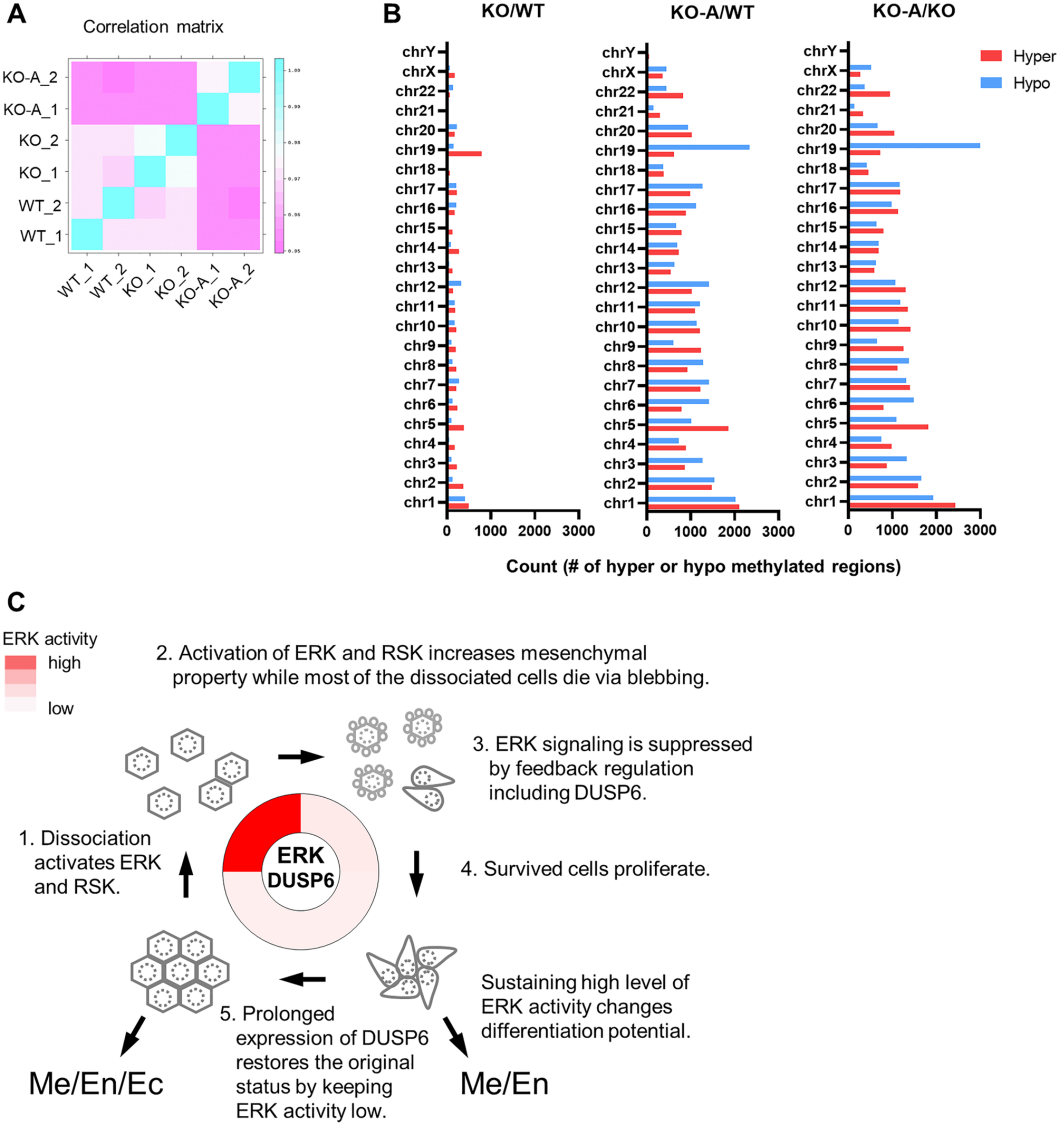
DNA methylation in *DUSP6* KO cells and modeling of cellular resilience. (**A**) Correlation matrix analysis of WT, KO, and KO-A. Samples were duplicated for analysis. (**B**) Comparison of hyper-methylated (red) or hypo-methylated (blue) regions between samples. (**C**) Proposed model of cellular resilience by ERK and DUSP6 feedback loop in response to cell dissociation in hPSCs. Well-formed cell–cell and cell–matrix connections maintain epithelial characteristics of hPSCs with a balanced differentiation propensity. Dissociation activates ERK and RSK, which increases mesenchymal property. Increased mesenchymal property may increase survival rate although most dissociated cells die. Survived cells proliferate and suppress ERK activity by prolonged expression of DUSP6. Without the suppression, cells sustaining high level of ERK activity changes differentiation potential. *Me* mesoderm, *En* endoderm, *Ec* ectoderm.

Differentiation of mESCs into the three germ layers is accompanied by epigenetic changes, including DNA methylation^42–44^. To investigate the effect of DUSP6 depletion on DNA methylation, we compared the methylation status of WT, *DUSP6* KO, and KO-A cells. The results showed that the DNA methylation pattern was significantly altered in *DUSP6* KO-A cells compared to WT and *DUSP6* KO cells, whereas no significant difference was observed between WT and *DUSP6* KO cells in the correlation matrix (Fig. 6A). Both hyper- and hypo-methylation sites were significantly altered throughout the genome of *DUSP6* KO-A cells (Fig. 6B). These results indicate that changes in DNA methylation accompany the altered differentiation propensity of DUSP6 KO-A cells.

## Discussion

Response to extracellular stimuli is vital for all living organisms. At the cellular level, this response can be measured by evaluating cell viability, morphology, and motility. Pluripotent stem cells provide another characteristic, differentiation propensity, although it is difficult to quantify. Previously, we developed a spontaneous differentiation method to quantify the differentiation propensity of pluripotent stem cells using 5% FBS and RNA-seq^8^. In this study, we demonstrated that the differentiation propensity can serve as an indicator of changes in cellular properties using this method.

Cell dissociation is a deadly stimulus for hPSCs because most of the dissociated single cells undergo cell death via ROCK-mediated membrane blebbing. Therefore, ROCK inhibitors have been widely used during passaging of hPSCs^9,10,45^. ROCK activity depends on the upstream effector Rho, a small GTPase^46^. Rho promotes long-term expansion and survival of hPSCs through the YAP/TAZ signaling pathway^47^. However, it is still unclear how other signaling pathways respond to cell dissociation.

ERK, first identified as an extracellular signal-regulated kinase, is an important molecule responsible for prominent cellular responses to extracellular stimuli^48,49^. In mESCs, ERK signaling is indispensable for both self-renewal and differentiation^16,17,50^. ERK signaling is also necessary for hPSCs as well^51^. hPSCs under steady state conditions have generally restrained ERK activity while addition of fresh growth factors transiently activates the signal^3,52^. Once self-renewal conditions have been re-established, ERK activity resets to basal level. The reason appears to be that long-lasting high ERK activity can initiate differentiation. Here we provide another example of ERK regulation and the effects of long-lasting high ERK activity.

We investigated the response of hPSCs to cell dissociation by assessing the gene expression profile of cells passaged using different methods. We observed that cell dissociation immediately activates the ERK signaling pathway in hPSCs. ERK inhibition using FAK and ROCK inhibitors showed that more than one mechanism is involved in ERK activation following cell dissociation. One is via FAK dissociating from the focal adhesion following cell dissociation, while the other is via ROCK. These two signals merge at MEK and ERK. ERK is one of the key mediators of epithelial-to-mesenchymal transition (EMT)^53^, and mesenchymal cells can survive better in adverse conditions, such as in detached conditions during embryonic development and carcinoma progression^54,55^. It is therefore plausible that rapid ERK activation following cell dissociation preserves cell viability by enhancing mesenchymal properties. RSK has been shown as a principal effector of the ERK pathway in invasive epithelial cells^24,56^. In our study, although enhanced ERK activity resulting from DUSP6 depletion produced a transformed outgrowth, it was not able to disrupt the colonies and cause a scattered mesenchymal phenotype like that observed in epithelial cells with enhanced ERK activity due to the overexpression of dominant active RAF^24,57^. This difference may be due to differences in the strength of cell–cell interactions or the level of ERK. FGF signaling in the primitive streak plays an important role in mesoderm formation during gastrulation in early mouse embryos^58,59^. The role of ERK-DUSP6 signaling in EMT of cancer cells is well established^60^. In endometrial adenocarcinoma, DUSP6 inhibits EMT^61^. In micropatterned cultures, hESCs exhibit high ERK activity in the boundary region of colonies where mesendoderm makers, such as SNAIL, are primarily expressed upon differentiation^62^. Therefore, it is possible that DUSP6 plays a role in increasing epithelial properties and decreasing mesenchymal properties by down-regulating basal ERK activity in the long term. Regarding lineage specification, it is likely that the mesenchymal properties and differentiation propensity of hPSCs towards the mesoderm are closely linked because transcription factors for mesenchymal properties, such as SNAIL and TWIST, also play roles in mesoderm formation. During early embryonic development, FGF signaling is indispensable for mesoderm induction and specification^63^, and it is also required for activin A-induced definitive endoderm formation in hESCs^64^. Consistent with this, *DUSP6* KO cells with increased basal ERK activity exhibited enhanced differentiation towards mesodermal and endodermal lineages.

We established two *DUSP6* KO cell lines, designated as *DUSP6* KO and KO-A, which originated from a single *DUSP6* KO clone. *DUSP6* KO cells had slightly increased p-ERK levels, while the increase in p-ERK level in *DUSP6* KO-A cells was much higher compared to WT cells. This difference between the two cell lines may be due to differences in the passaging methods used after selection of the *DUSP6* KO clone. *DUSP6* KO-A was established by mechanical passaging, whereas *DUSP6* KO was by EDTA passaging. One possibility is that EDTA passaging activates other feedback proteins, while mechanical passaging does not. During subculture, the elevated ERK levels of mechanically passaged cells might have altered their DNA methylation profile similarly to that seen in *DUSP6* KO-A. A previous study showed that in colon cancer cells, changes in MEK/ERK signaling are accompanied by alterations in DNA methylation^65^. Given that the differentiation of ESCs is accompanied by DNA methylation and demethylation that eventually leads to the formation of cell- or tissue-specific methylation patterns^42,44^, it appears that the altered DNA methylation in *DUSP6* KO-A may affect their differentiation propensity.

Although programmed cell death such as apoptosis, anoikis, and ferroptosis may occur during hPSC dissociation, the exact mechanism by which cell death occurs during hPSC dissociation remains unclear. Recent studies demonstrated that dissociation can activate mitochondrial-associated apoptosis in hESCs through the PKA/p53/Bax signaling pathway^66^, and cytoskeleton disruption can lead to ferroptosis accompanied by the production of reactive oxygen species through iron accumulation in hESCs^67^. Anoikis, a type of apoptosis caused by the disruption of cell–cell and cell–matrix attachment^68,69^, can also occur. Cell death in cultured hPSCs following single-cell dissociation can be considered a form of anoikis because the RAS/MAPK signaling pathway is involved in anoikis resistance and acts as a pro-survival signal^70–72^. The simultaneous activation of ERK and ROCK following cell dissociation indicates that disruption of cell–cell and cell–matrix attachment in hPSCs activates pro-survival and pro-apoptotic signals simultaneously. The pro-survival signal is rapidly suppressed through feedback regulations, including DUSP6. However, the feedback by DUSP6 lasts even after termination of the ERK signaling, lowering the basal level of the signal to restore the original cellular properties. This cellular capacity for survival and restoration of the original status can be considered as cellular resilience^73^. If this resilience does not work properly, hPSCs could be spontaneously transformed into cancer cells with a tilted differentiation propensity, as observed in *DUSP6* KO-A cells. Therefore, DUSP6 plays a role in maintaining the memory of previous experiences that helps the cells to restore their original status by acting as a long-term feedback regulator of basal ERK activity. Based on these observations, we propose a resilience model for the survival and restoration of hPSCs after the dissociation stimulus. In this model, (1) cell dissociation activates ERK, (2) the activation of ERK signaling following cell dissociation increases the mesenchymal properties, which in turn increases cell survival at the cost of differentiation potential, (3) ERK signaling is suppressed by feedback regulation, including DUSP6, (4) surviving cells undergo proliferation, and (5) cells recover their original differentiation potential by lowering the basal ERK activity. If the ERK signal persists due to improper feedback regulation, DNA methylation pattern changes, followed by an irreversible change in the differentiation potential (Fig. 6C).

Accumulating evidence suggests that passaging methods affect genomic stability in hPSCs, thus, affecting the quality and clinical applications of these cells^74,75^. In good manufacturing practice (GMP) conditions, however, mechanical passaging is not possible because minimal exposure requires the use of flasks rather than dishes. Therefore, genomic stability in cells that have been passaged using EDTA or enzymes should be analyzed carefully over a long period. Proper regulation of the ERK activity appears to be necessary for genomic stability because ERK 1/2 depletion has been shown to cause genomic abnormalities in mESCs^16^. The effect of long-term treatment with the ROCK inhibitor on preventing dissociation-induced cell death needs further consideration because ROCK is involved in mitotic spindle organization^76^.

In summary, our findings showed that the memory retention feedback of ERK signaling by DUSP6 is required for cellular resilience in hPSCs, and provide insights into the cellular response towards extracellular stimuli.

## Methods

### Cell culture

Human pluripotent stem cells (hPSCs) were grown in the TeSR-E8 medium (Stem Cell Technologies). Vitronectin (VTN-N, Thermo Fisher) was used as the matrix. Cells were passaged once every 5–7 days using EDTA for chemical passaging or EZPassage (Thermo Fisher) for mechanical passaging. EDTA was added for 3 min. The human induced pluripotent stem cell line, hFSiPS1 (KSCBi002-A), and the human embryonic stem cell line, SNUhES31 (SNUe007-A, established by Seoul National University), used in this study, were obtained from the Korea National Stem Cell Bank^77,78^.

### Cell harvest

Cells were harvested using a cell scraper or cell dissociation solution after washing twice with DPBS. Cells were transferred into 1.5 mL centrifuge tubes and centrifuged and after removing DPBS cells were lysed by adding lysis solution containing phosphatase inhibitors. For the comparison of p-ERK levels according to the harvesting methods, cells were harvested as follow: for scrapper (Scrp) condition, cells were harvested using a scrapper (SPL); for cell dissociation solutions such as EDTA, ReLeSR (Stem Cell Technologies), TrypLE (Thermo Fisher) and Dispase (Stem Cell Technologies) cells were incubated in the dissociation solution and harvested; for Direct condition, cells were directly lysed on culture dishes. For dissociation solutions, cells were treated with each dissociation solution as follow: EDTA for 4 min, ReLeSR for 4 min, TrypLE for 4 min, Dispase for 15 min at 37 °C.

### RNA-seq

Total RNA was isolated from hPSCs using the Maxwell RSC RNA FFPE kit and automated Maxwell RSC Instrument (Promega) according to the manufacturer’s instructions. RNA concentration was determined using Quant-IT RiboGreen (Invitrogen). RNA integrity was assessed using the TapeStation RNA ScreenTape (Agilent). Only high-quality RNA with RIN greater than 7.0 was used for RNA library construction.

Library was prepared using 1 µg of total RNA from each sample using the Illumina TruSeq Stranded mRNA Sample Prep Kit (Illumina). The first step in the workflow involves purifying the poly-A-containing mRNA molecules using poly‐T‐attached magnetic beads. Following purification, mRNA was fragmented into small pieces using divalent cations at elevated temperatures. The cleaved RNA fragments were reverse transcribed to first-strand cDNA using SuperScript II reverse transcriptase (#18064014; Invitrogen) and random primers. This was followed by second-strand cDNA synthesis using DNA polymerase I, RNase H, and dUTPs. These cDNA fragments then go through an end repair process, the addition of a single ‘A’ base, and then ligation of the adapters. The products were purified and enriched by PCR to generate the final cDNA library.

The libraries were quantified using the KAPA Library Quantification kit for Illumina Sequencing platforms according to the qPCR Quantification Protocol Guide (KAPA BIOSYSTEMS) and qualified using TapeStation D1000 ScreenTape (Agilent Technologies). Indexed libraries were then subjected to paired-end (2 × 151 bp) sequencing using Illumina HiSeqXten (Illumina) at Macrogen. Some samples were sequenced on the Illumina NovaSeq6000 platform (150 bp paired-end) following manufacturer’s protocol. Image analysis was performed using the NovaSeq6000 control software version 1.3.1, and output base calling data were de-multiplexed with bcl2fastq version v2.20.0422 to generate fastQ files. Sequencing on the NovaSeq6000 platform was performed at Theragen and Macrogen.

The relative abundance of genes was measured in terms of read counts using Cufflinks. We performed statistical analysis to identify differentially expressed genes using the estimates of abundance for each gene in the samples. Genes with one or more than zero read count values in the samples were excluded. To facilitate log2 transformation, one was added to each read count value of the filtered genes. Filtered data were log2-transformed and subjected to TMM normalization. Statistical significance was determined by exactTest using edgeR and fold change, in which the null hypothesis was that no difference exists among groups. The false discovery rate (FDR) was controlled by adjusting the p-value using the Benjamini–Hochberg algorithm. For the DEG set, hierarchical clustering analysis was performed using complete linkage and Euclidean distance as similarity measures. Gene-enrichment, functional annotation analysis, and pathway analysis for differentially expressed genes were performed using the online platform Gene Ontology (www.geneontology.org/).

### Quantitative real-time PCR

Total RNA was isolated using the same method used for RNA-seq. Reverse transcription was performed with 2 µg of total RNA using the RNA to cDNA EcoDry Premix (Takara). Quantitative real-time PCR was performed using Taqman^®^ Primer and Probe sets (Thermo Fisher). ACTB was used as the internal control. PCR was performed using QuantStudio 6 Flex (Life Technologies). Fold changes were calculated using the ∆∆CT method^79^.

### Western blotting

Whole cell extract was prepared using the radioimmunoprecipitation assay (RIPA) lysis buffer (Thermo Fisher) supplemented with protease inhibitors (Roche) and phosphatase inhibitors (Sigma). Protein concentration in the lysates was determined using the BCA Protein Assay Reagent (Pierce). Protein (15–20 µg) samples were then separated on 4–12% Bis–Tris Plus gels (Thermo Fisher) and transferred to PVDF membranes (Millipore) using the iBind™ Flex Western Starter Kit (Thermo Fisher). Membranes were then incubated with primary antibodies and the appropriate secondary antibodies. Quantity of the loaded proteins was validated using anti-α-tubulin or anti-β-actin antibodies (Sigma, 1:2000). The chemiluminescence signal was developed using Amersham ECL Prime Western Blotting Detection Reagent (GE Healthcare Life Sciences) following the manufacturer’s protocol and captured by an Amersham Imager 600 (GE Healthcare Life Sciences) using the automatic mode.

### Isolation of nuclei

Nuclei were isolated from cultured cells as described by Nabbi et al.^80^. Briefly, dishes containing 90% confluent cells were washed with ice-cold PBS. Cells were then scraped off in 1 mL PBS and collected in a 1.5 mL microcentrifuge tube on ice. Cells were pelleted by centrifugation and resuspended in 0.1% NP-40 containing ice-cold PBS. Cells were triturated five times on ice using a P1000 micropipette tip. The lysed cell suspension was centrifuged for 5–10 s at 10,000 *rpm*. The supernatant was then transferred to a new tube and used as the cytoplasmic fraction. The pellet was resuspended in PBS containing 0.1% NP-40 and centrifuged for 5–10 s at 10,000 *rpm*. After discarding the supernatant, the pellet was resuspended in 1 × Laemmli sample buffer, and used as the nuclear fraction.

### Transfection with CRISPR/Cas9

sgRNAs transfection into hFSiPS1 cells was performed by electroporation (Amax P3 Primary Cell 4D-Nucleofector^®^ X Kit) according to the manufacturer's instructions. Briefly, 10 µg of Cas9 protein (Toolgen) was mixed with 2 µg of sgRNA (Toolgen) for transfecting 1.5 × 10^6^ cells. To reduce the damage due to DUSP6 depletion, cells were maintained in knockout serum replacement-containing medium (KSR, Thermo Fisher). Mitomycin C-inactivated mouse embryonic fibroblasts (STO) were used as feeder cells. After electroporation, the cells were plated on STO-coated 96 well plates with a limit dilution of single cell per well. To increase the survival, single cells were treated with CloneR (Stem Cell Technologies) for 12 h after electroporation. When the cell clumps were formed in approximately 12 days, we harvested half of the colony, isolated genomic DNA, and amplified the target region. The amplified DNA fragments were analyzed by Sanger sequencing (Bioneer). KO cells having early terminating mutations on both chromosomes were selected.

### Cell survival assay

Cells were seeded at a density of 5 × 10^4^ cells per well on 96 well plate coated with Vitronectin (VTN-N, Thermo Fisher). Survival rate was estimated by CCK-8 kit (Dojindo) using Eon microplate spectrophotometer (BioTeK).

### Real-time cell growth assay

The attachment and growth of hPSCs were determined using the xCELLigence real-time cell analyzer (RTCA) S16 (Agilent). Briefly, 150 µL of TeSR-E8 medium was placed in each well of the E-plate 16 (gold-microelectrode array integrated E-plate, Agilent) that was pre-coated with VTN-N (Thermo Fisher). Cells were dissociated using EDTA for 5 min and pipetting five times with a 1 mL micropipette. Cell number was determined using the LUNA FX7 automated cell counter (Logos Biosystems). Cells were then diluted to 3 × 10^4^, 4 × 10^4^, 5 × 10^4^, and 6 × 10^4^ in 50 µL for four replicates. The E-plates were incubated at room temperature for 30 min in a laminar flow cabinet and then placed on the RTCA Station located in an incubator at 37 °C for continuous impedance recording.

### Spontaneous differentiation using 5% FBS

For spontaneous differentiation, the medium was changed to DMEM/F12 containing 0.1 mM 2-mercaptoethanol, 1% NEAA, 1% antibiotic–antimycotic, 0.1% Mycozap Plus-PR, and 5% fetal bovine serum (FBS). The medium was changed every 2 days. Cells were harvested at each time point and analyzed using RNA-seq.

### Teratoma assay

Immune deficient NOD/SCID mice were used for teratoma assay. Cell suspensions (1 × 10^6^ cells in 100 µL) were injected subcutaneously using a sterile 1 mL syringe with a 23 G needle. Teratoma formation was monitored visually for 8 weeks. After 8 weeks, the mice were euthanasia by CO2 asphyxiation and teratomas were carefully excised from the surrounding tissue. After taking the pictures, teratomas were cut into two pieces. One piece was fixed in Bouin’s solution followed by paraffin sectioning, while the other piece was kept at − 80 °C. Sections (5 µm thick) were cut (Leica RM2255) and stored at 4 °C prior to staining or immunohistochemistry. Hematoxylin and eosin staining was performed according to standard protocols. Teratomas were assessed by a board-certified pathologist at Kangwon National University.

Since histological assessments of naturally occurring human teratomas are routinely performed, the composition of each teratoma was estimated semi-quantitatively by visual inspection of the H&E-stained sections in a blinded fashion. Differentiated elements in the teratomas were classified into ectodermal (skin, neural, etc.), endodermal (glandular structures), and mesodermal (cartilage, bone) lineage as a percentage of the entire tissue. All experimental procedures involving animals were conducted by GHbio.

### Cardiomyocyte differentiation of hiPSCs

Cardiomyocyte differentiation of hiPSCs was performed by T&R Biofab. Briefly, the WT and *DUSP6* KO hiPSC lines were maintained in StemMACS iPS-BREW XF media (Miltenyi Biotec) on Matrigel (Corning). For cardiomyocyte differentiation, hiPSCs were seeded onto hPSC-certified Matrigel-coated cell culture dishes (Eppendorf) at 140,000 cells/cm^2^. Y-27632 (5 μM) (Tocris) was added for the first 24 h after passaging. The medium was changed daily and hiPSCs were allowed to grow for 3–4 days until they reached 90% confluence. At day 0, cells were treated with 6 μM of CHIR99021 (Tocris) in cardiomyocyte differentiation medium (CDM; RPMI1640 [Thermo Fisher] supplemented with bovine serum albumin [BSA, Sigma-Aldrich] and ascorbic acid [Sigma-Aldrich]). After 48 h of incubation, the medium was changed to CDM supplemented with 2 μM of C59, a Wnt inhibitor (Stemgent Inc.), and further incubated for 48 h. On day 5, the medium was replaced with fresh CDM, and subsequently replaced with fresh medium every other day. Contracting cells began to appear on days 8–10. From days 10–15, CDM containing l-lactic acid was used to metabolically select and purify hiPSC-derived cardiomyocytes. Images were analyzed using the Eclipse-Ti2 fluorescence microscope (Nikon).

### Flow cytometry

hPSC-derived cardiomyocytes were fixed with 4% PFA/permeabilization solution (BD Bioscience) for 15 min. Cardiomyocytes were stained using anti-TNNT2 (Abcam) and anti-myosin 4 antibodies (Thermo Fisher) for 30 min at 4 °C. Subsequently, cells were incubated with fluorescein-conjugated secondary antibodies for 30 min and then analyzed using the SH800S Cell Sorter with Cell sorter software version 2.1.5 (Sony Biotechnology).

### Immunocytochemistry

Cells were fixed with 4% PFA (Wako) for 20 min and permeabilized with 0.2% (v/v) Triton X-100 (Sigma Aldrich) in 1 × PBS for 10 min. Cells were blocked with 1% (v/v) BSA (Sigma Aldrich) in 1 × PBS for 30 min and then incubated with primary antibodies (listed in the Key Resource Table) overnight at 4 °C. Cells were subsequently incubated with fluorescein-conjugated secondary antibodies for 1 h. Images were acquired using a confocal microscope (FV3000, Olympus).

### Confocal fluorescence microscopy

Confocal fluorescent images were obtained using the confocal microscope FV3000 (Olympus) with 10 × and 40 × objectives at the microscopy facility of the KNIH. Sequential excitation with 488, 594, and 405 nm was achieved by a diode-type laser. After sequential excitation, green, red, and blue fluorescent images were saved using the FV31S-SW software version 2.3.1.162.

### Analysis of DNA methylation

Genomic DNA was extracted from hPSCs using the Maxwell RSC Cell DNA Purification Kit with an automated Maxwell RSC Instrument (Promega), according to manufacturer’s instructions. For each sample, 1 µg of genomic DNA was bisulfite-converted and enriched using the SureSelect Methyl-Seq Kit (Agilent Technologies). Briefly, the isolated genomic DNA was sheared to 150–200 bp, hybridized, and captured on streptavidin beads. The captured gDNA was eluted, and unmethylated C residues were converted to uracil using bisulfite. Di-tagged DNA was enriched by PCR and sequenced using the HiSeq platform. After sequencing, raw sequence reads were filtered based on their quality. The trimmed reads were mapped to the reference genome using BSMAP, which is based on SOAP (Short Oligo Alignment Program). Only uniquely mapped reads were selected for sorting and indexing, and PCR duplicates were removed using SAMBAMBA (v0.5.9). The methylation ratio of every single cytosine located within the target region was extracted from the mapping results using ‘methylatio.py’ script in BSMAP. The coverage profiles were calculated as the number of C/effective CT counts for each cytosine in CpG, CHH, and CHG. Each cytosine locus in CpG, CHH, and CHG was annotated using the table browser function of the UCSC Genome Browser. Differentially methylated CpGs between groups were calculated using the difference between the two groups (delta mean), the independent t-test, and hierarchical clustering. The filtering criteria were as follows: hypermethylated = delta mean ≥ 0.2 and p-value < 0.05, hypomethylated = delta mean ≤ − 0.2 and p-value < 0.05.

## Regulatory and institutional review

All hPSC experiments were approved by Institutional Review Board of the Korea National Institute of Health. All experimental procedures involving animals were conducted in accord with experimental protocols approved by the Institutional Animal Care and Use Committee of the Korea Research Institute of Bioscience and Biotechnology. All protocols were reported in accordance with ARRIVE guidelines.

### Statistical analyses

Statistical analyses were conducted using Prism software (GraphPad 9.5.1). For pairwise and two independent group comparison two-tailed t-test were used. Statistically significant p-values are indicated in the figures. A p-value < 0.05 was considered statistically significant. Data are expressed as the mean ± SEM of at least three independent experiments.

## Supplementary Information

Supplementary Information.

## Data Availability

Cell lines generated in this study can be obtained from the Korea National Stem Cell Bank (Email: nscb@korea.kr) after completing the Materials Transfer Agreement. The RNA-seq datasets generated in the present study are available at the GEO repository with accession number GSE205656. The data that support the findings of this study are included here and in the Supplementary Information are also available on demand.

## References

[1] Takahashi K, Yamanaka S (2006). Induction of pluripotent stem cells from mouse embryonic and adult fibroblast cultures by defined factors. Cell.

[2] Thomson JA (1998). Embryonic stem cell lines derived from human blastocysts. Science.

[3] Chen G (2011). Chemically defined conditions for human iPSC derivation and culture. Nat. Methods.

[4] Nakagawa M (2014). A novel efficient feeder-free culture system for the derivation of human induced pluripotent stem cells. Sci. Rep..

[5] Vuoristo S (2013). A novel feeder-free culture system for human pluripotent stem cell culture and induced pluripotent stem cell derivation. PLoS One.

[6] Ludwig TE (2006). Feeder-independent culture of human embryonic stem cells. Nat. Methods.

[7] Yamamoto T (2018). Differentiation potential of Pluripotent Stem Cells correlates to the level of CHD7. Sci. Rep..

[8] Yoo DH (2020). Simple differentiation method using FBS identifies DUSP6 as a marker for fine-tuning of FGF-ERK signaling activity in human pluripotent stem cells. Biochem. Biophys. Res. Commun..

[9] Watanabe K (2007). A ROCK inhibitor permits survival of dissociated human embryonic stem cells. Nat. Biotechnol..

[10] Chen G, Hou Z, Gulbranson DR, Thomson JA (2010). Actin-myosin contractility is responsible for the reduced viability of dissociated human embryonic stem cells. Cell Stem Cell.

[11] Walker A (2010). Non-muscle myosin II regulates survival threshold of pluripotent stem cells. Nat. Commun..

[12] Toh YC, Xing J, Yu H (2015). Modulation of integrin and E-cadherin-mediated adhesions to spatially control heterogeneity in human pluripotent stem cell differentiation. Biomaterials.

[13] Li D (2010). Integrated biochemical and mechanical signals regulate multifaceted human embryonic stem cell functions. J. Cell Biol..

[14] Chowdhury F (2010). Material properties of the cell dictate stress-induced spreading and differentiation in embryonic stem cells. Nat. Mater..

[15] Göke J, Chan YS, Yan J, Vingron M, Ng HH (2013). Genome-wide kinase-chromatin interactions reveal the regulatory network of ERK signaling in human embryonic stem cells. Mol. Cell.

[16] Chen H (2015). Erk signaling is indispensable for genomic stability and self-renewal of mouse embryonic stem cells. Proc. Natl. Acad. Sci. U. S. A..

[17] Kunath T (2007). FGF stimulation of the Erk1/2 signalling cascade triggers transition of pluripotent embryonic stem cells from self-renewal to lineage commitment. Development.

[18] Grünert S, Jechlinger M, Beug H (2003). Diverse cellular and molecular mechanisms contribute to epithelial plasticity and metastasis. Nat. Rev. Mol. Cell Biol..

[19] Hansen SH (2000). Induced expression of Rnd3 is associated with transformation of polarized epithelial cells by the Raf-MEK-extracellular signal-regulated kinase pathway. Mol. Cell Biol..

[20] Janda E (2002). Ras and TGF[beta] cooperatively regulate epithelial cell plasticity and metastasis: Dissection of Ras signaling pathways. J. Cell Biol..

[21] Lehmann K (2000). Raf induces TGFbeta production while blocking its apoptotic but not invasive responses: A mechanism leading to increased malignancy in epithelial cells. Genes Dev..

[22] Dowd S, Sneddon AA, Keyse SM (1998). Isolation of the human genes encoding the pyst1 and Pyst2 phosphatases: characterisation of Pyst2 as a cytosolic dual-specificity MAP kinase phosphatase and its catalytic activation by both MAP and SAP kinases. J. Cell Sci..

[23] Li C, Scott DA, Hatch E, Tian X, Mansour SL (2007). Dusp6 (Mkp3) is a negative feedback regulator of FGF-stimulated ERK signaling during mouse development. Development.

[24] Doehn U (2009). RSK is a principal effector of the RAS-ERK pathway for eliciting a coordinate promotile/invasive gene program and phenotype in epithelial cells. Mol. Cell.

[25] Ekerot M (2008). Negative-feedback regulation of FGF signalling by DUSP6/MKP-3 is driven by ERK1/2 and mediated by Ets factor binding to a conserved site within the DUSP6/MKP-3 gene promoter. Biochem. J..

[26] Arkell RS (2008). DUSP6/MKP-3 inactivates ERK1/2 but fails to bind and inactivate ERK5. Cell Signal.

[27] Slack-Davis JK (2007). Cellular characterization of a novel focal adhesion kinase inhibitor. J. Biol. Chem..

[28] Aoki K (2016). A RhoA and Rnd3 cycle regulates actin reassembly during membrane blebbing. Proc. Natl. Acad. Sci. U. S. A..

[29] Maillet M (2008). DUSP6 (MKP3) null mice show enhanced ERK1/2 phosphorylation at baseline and increased myocyte proliferation in the heart affecting disease susceptibility. J. Biol. Chem..

[30] Mansour SJ (1994). Transformation of mammalian cells by constitutively active MAP kinase kinase. Science.

[31] Robinson MJ, Stippec SA, Goldsmith E, White MA, Cobb MH (1998). A constitutively active and nuclear form of the MAP kinase ERK2 is sufficient for neurite outgrowth and cell transformation. Curr. Biol..

[32] Murphy LO, Blenis J (2006). MAPK signal specificity: The right place at the right time. Trends Biochem. Sci..

[33] Hamilton WB, Brickman JM (2014). Erk signaling suppresses embryonic stem cell self-renewal to specify endoderm. Cell Rep..

[34] Li Z, Theus MH, Wei L (2006). Role of ERK 1/2 signaling in neuronal differentiation of cultured embryonic stem cells. Dev. Growth Differ..

[35] Patel AL, Shvartsman SY (2018). Outstanding questions in developmental ERK signaling. Development.

[36] Pokrass MJ (2020). Cell-cycle-dependent ERK signaling dynamics direct fate specification in the mammalian preimplantation embryo. Dev. Cell.

[37] Simon CS, Rahman S, Raina D, Schröter C, Hadjantonakis AK (2020). Live visualization of ERK activity in the mouse blastocyst reveals lineage-specific signaling dynamics. Dev Cell.

[38] Sulzbacher S, Schroeder IS, Truong TT, Wobus AM (2009). Activin A-induced differentiation of embryonic stem cells into endoderm and pancreatic progenitors-the influence of differentiation factors and culture conditions. Stem Cell Rev. Rep..

[39] Huang TS (2015). A regulatory network involving β-catenin, e-cadherin, PI3k/Akt, and slug balances self-renewal and differentiation of human pluripotent stem cells in response to Wnt signaling. Stem Cells.

[40] Kunisada Y, Tsubooka-Yamazoe N, Shoji M, Hosoya M (2012). Small molecules induce efficient differentiation into insulin-producing cells from human induced pluripotent stem cells. Stem Cell Res..

[41] Sances S (2016). Modeling ALS with motor neurons derived from human induced pluripotent stem cells. Nat. Neurosci..

[42] Isagawa T (2011). DNA methylation profiling of embryonic stem cell differentiation into the three germ layers. PLoS One.

[43] Mohn F (2008). Lineage-specific polycomb targets and de novo DNA methylation define restriction and potential of neuronal progenitors. Mol. Cell.

[44] Meissner A (2008). Genome-scale DNA methylation maps of pluripotent and differentiated cells. Nature.

[45] Harb N, Archer TK, Sato N (2008). The Rho-Rock-Myosin signaling axis determines cell-cell integrity of self-renewing pluripotent stem cells. PLoS One.

[46] Riento K, Ridley AJ (2003). Rocks: Multifunctional kinases in cell behaviour. Nat. Rev. Mol. Cell Biol..

[47] Ohgushi M, Minaguchi M, Sasai Y (2015). Rho-signaling-directed YAP/TAZ activity underlies the long-term survival and expansion of human embryonic stem cells. Cell Stem Cell.

[48] Boulton TG, Gregory JS, Cobb MH (1991). Purification and properties of extracellular signal-regulated kinase 1, an insulin-stimulated microtubule-associated protein 2 kinase. Biochemistry.

[49] Boulton TG (1990). An insulin-stimulated protein kinase similar to yeast kinases involved in cell cycle control. Science.

[50] Yao Y (2003). Extracellular signal-regulated kinase 2 is necessary for mesoderm differentiation. Proc. Natl. Acad. Sci. U. S. A..

[51] Dalton S (2013). Signaling networks in human pluripotent stem cells. Curr. Opin. Cell Biol..

[52] Singh AM (2012). Signaling network crosstalk in human pluripotent cells: A Smad2/3-regulated switch that controls the balance between self-renewal and differentiation. Cell Stem Cell.

[53] Shin S (2019). ERK2 regulates epithelial-to-mesenchymal plasticity through DOCK10-dependent Rac1/FoxO1 activation. Proc. Natl. Acad. Sci. U. S. A..

[54] Nieto MA (2013). Epithelial plasticity: A common theme in embryonic and cancer cells. Science.

[55] Nieto MA, Huang RY, Jackson RA, Thiery JP (2016). EMT: 2016. Cell.

[56] Čáslavský J, Klímová Z, Vomastek T (2013). ERK and RSK regulate distinct steps of a cellular program that induces transition from multicellular epithelium to single cell phenotype. Cell Signal.

[57] Lehmann K (2000). Raf induces TGFbeta production while blocking its apoptotic but not invasive responses: A mechanism leading to increased malignancy in epithelial cells. Genes Dev..

[58] Arnold SJ, Robertson EJ (2009). Making a commitment: Cell lineage allocation and axis patterning in the early mouse embryo. Nat. Rev. Mol. Cell Biol..

[59] Ciruna B, Rossant J (2001). FGF signaling regulates mesoderm cell fate specification and morphogenetic movement at the primitive streak. Dev. Cell.

[60] Olea-Flores M (2019). Extracellular-signal regulated kinase: A central molecule driving epithelial-mesenchymal transition in cancer. Int. J. Mol. Sci..

[61] Fan MJ (2019). Dusp6 inhibits epithelial-mesenchymal transition in endometrial adenocarcinoma via ERK signaling pathway. Radiol. Oncol..

[62] Warmflash A, Sorre B, Etoc F, Siggia ED, Brivanlou AH (2014). A method to recapitulate early embryonic spatial patterning in human embryonic stem cells. Nat. Methods.

[63] Kumar V, Goutam RS, Park S, Lee U, Kim J (2021). Functional roles of FGF signaling in early development of vertebrate embryos. Cells.

[64] Sui L, Mfopou JK, Geens M, Sermon K, Bouwens L (2012). FGF signaling via MAPK is required early and improves Activin A-induced definitive endoderm formation from human embryonic stem cells. Biochem. Biophys. Res. Commun..

[65] Lu R (2007). Inhibition of the extracellular signal-regulated kinase/mitogen-activated protein kinase pathway decreases DNA methylation in colon cancer cells. J. Biol. Chem..

[66] Zhang L (2018). Activated mitochondrial apoptosis in hESCs after dissociation involving the PKA/p-p53/Bax signaling pathway. Exp. Cell Res..

[67] Babaei-Abraki S, Karamali F, Nasr-Esfahani MH (2022). Monitoring the induction of ferroptosis following dissociation in human embryonic stem cells. J. Biol. Chem..

[68] Frisch SM, Francis H (1994). Disruption of epithelial cell-matrix interactions induces apoptosis. J. Cell Biol..

[69] Adeshakin FO (2021). Mechanisms for modulating Anoikis resistance in cancer and the relevance of metabolic reprogramming. Front. Oncol..

[70] Guo YJ (2020). ERK/MAPK signalling pathway and tumorigenesis. Exp. Ther. Med..

[71] de Sousa Mesquita AP, de Araújo Lopes S, Pernambuco Filho PCA, Nader HB, Lopes CC (2017). Acquisition of anoikis resistance promotes alterations in the Ras/ERK and PI3K/Akt signaling pathways and matrix remodeling in endothelial cells. Apoptosis.

[72] Cao L (2014). Mitogen-activated protein kinase pathway is pivotal for anoikis resistance in metastatic hepatoma cells. Mol. Med. Rep..

[73] Smirnova L, Harris G, Leist M, Hartung T (2015). Cellular resilience. Altex.

[74] Garitaonandia I (2015). Increased risk of genetic and epigenetic instability in human embryonic stem cells associated with specific culture conditions. PLoS One.

[75] Ohnuma K (2014). Enzyme-free passage of human pluripotent stem cells by controlling divalent cations. Sci. Rep..

[76] Närvä E (2017). A strong contractile actin fence and large adhesions direct human pluripotent colony morphology and adhesion. Stem Cell Rep..

[77] Uhm KO (2017). Generation of human induced pluripotent stem cell lines from human dermal fibroblasts using a non-integration system. Stem Cell Res..

[78] Lee JY, Lee DY, Choi YS, Lee KJ, Kim YO (2011). Registration of human embryonic stem cell lines: Korea, 2010. Osong Public Health Res. Perspect..

[79] Livak KJ, Schmittgen TD (2001). Analysis of relative gene expression data using real-time quantitative PCR and the 2−ΔΔCT method. Methods.

[80] Nabbi A, Riabowol K (2015). Rapid isolation of nuclei from cells in vitro. Cold Spring Harb. Protoc..

