# Supplementary material for: DUSP6 is a memory retention feedback regulator of ERK signaling for cellular resilience of human pluripotent stem cells in response to dissociation
Source: Sci Rep. 2023 Apr 7;13:5683. doi: 10.1038/s41598-023-32567-8 (PMC10082014; doi:10.1038/s41598-023-32567-8)
Supplement: Supplementary file 1 — Supplementary Information. [file 41598_2023_32567_MOESM1_ESM.pdf]

Figure S1

A

```
ATGATAGATACGCTCAGACCCGTGCCCTTCGCGTCGGAAATGGCGATCAGCAAGACGGTGGCGTGGCTCAACGAGCAGCTGGAGCTGGGCAACG
AGCGGCTGCTGCTGATGGACTGCCGGCCCAGGAGCTATACGAGTCGTCGCACATCGAGTCGGCCATCAACGTGGCCATCCCGGGCATCATGCTG
CGGCGCCTGCAGAAGGGTAACCTGCCGGTGCGCGCGCTCTTCACGCGCGGCGAGGACCGGGACCGCTTCACCCGGCGCTGTGGCACCGACACAG
TGGTGCTCTACGACGAGAGCAGCAGCGACTGGAACGAGAATACGGGCGGCGAGTCGGTGCTCGGGCTGCTGCTCAAGAAGCTCAAGGACGAGGG
CTGCCGGGCGTTCTACCTGGAAG
```

B

				Mismatches		
	Target (5′ – 3′)	Direction	Cutting position	1 bp	2 bp	3 bp
RG1	ATTTCCGACGCGAAGGGCACGGG	–		0	0	0
RG2	GATCGCCATTTCCGACGCGAAGG	–		0	0	0
RG3	AGCGCCGGGTGAAGCGGTCCCGG	–		0	0	1
RG4	CGAGAATACGGGCGGCGAGTCGG	+	89351706	0	0	1

C

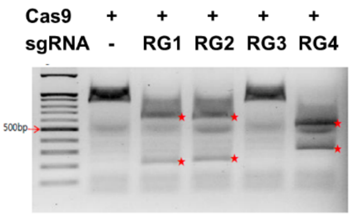

PCR amplification of human DUSP6 locus

```
Primer : DUSP6_F   GTAGAAAAGGCCGTGTGCTC
         DUSP6_R   CTGGGTTCAGACTTGGCATT
Amplicon size : 779
```

Gene	Total size	RG	Fragment	
Human DUSP6	779	RG1	195	584
		RG2	202	577
		RG3	422	357
		RG4	505	274

Figure S1. Design and validation of sgRNAs for *DUSP6* knockout. (A) Sequence of exon 1 of the *DUSP6* gene. (B) Sequences of sgRNAs (RG1, RG2, RG3, and RG4). Target sequences were screened using the CRISPR RGEN tool. (C) Validation of the selected sgRNAs was performed using the T7E1 assay. The 779 bp fragment of the *DUSP6* gene was amplified with DUSP6_F and DUSP6_R primers. For oligonucleotide sequences, see Key Resource Table. The amplified product was treated with each sgRNA and Cas9 protein and then subjected to T7E1 assay.

Figure S2

A

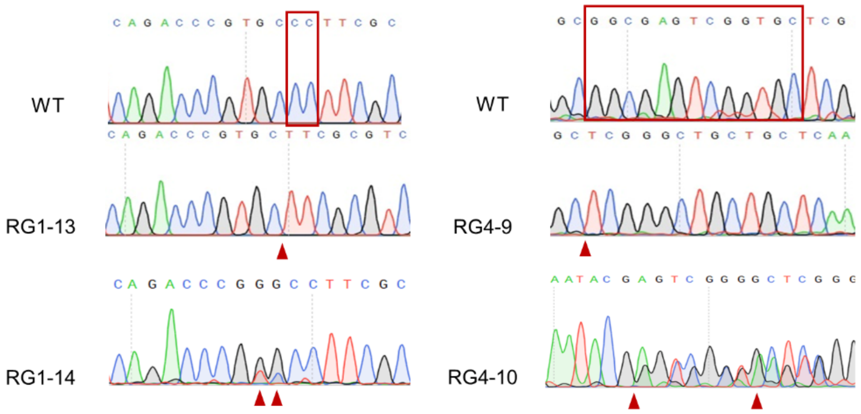

B

```
                   M   I   D   T   L   R   P   V   P   F   A   S   E   M   A     15
DUSP6_WT          ATG ATA GAT ACG CTC AGA CCC GTG CCC TTC GCG TCG GAA ATG GCG
DUSP6KO_RG1-13    ATG ATA GAT ACG CTC AGA CCC GTG C** TTC GCG TCG GAA ATG GCG   (-2)
                  ATG ATA GAT ACG CTC AGA CCC GTG C** TTC GCG TCG GAA ATG GCG   (-2)
DUSP6KO_RG1-14    ATG ATA GAT ACG CTC AGA CCC G*G CCC TTC GCG TCG GAA ATG GCG   (-1)
                  ATG ATA GAT ACG CTC AGA CCC GTG *CC TTC GCG TCG GAA ATG GCG   (-1)

                   D   W   N   E   N   T   G   G   E   S   V   L   G   L   L    118
DUSP6_WT          GAC TGG AAC GAG AAT ACG GGC GGC GAG TCG GTG CTC GGG CTG CTG
DUSP6KO_RG4-9     GAC TGG AAC GAG AAT ACG GGC *** *** *** *** *TC GGG CTG CTG   (-13)
                  GAC TGG AAC GAG AAT ACG GGC *** *** *** *** *TC GGG CTG CTG   (-13)
DUSP6KO_RG4-10    GAC TGG AAC GAG AAT ACG *** *** *AG TCG GTG CTC GGG CTG CTG   (-7)
                  GAC TGG AAC GAG AAT ACG GGC GGC GAA GTC GGT GCT CGG GCT GCT   (+1)
```

C

```
DUSP6_WT          MIDTLRPVPFASEMAISKTVAWLNEQLELGNERLLLMDCRPQELYESSHHIESAINVAIPGIML    63
DUSP6KO_RG1-13    MIDTLRPVLRVGNGDQQDGGVAQRAAGAGQRAAAADGLPAAGAIRVVAHRVGHQRGHPGHHAAAPAEG*
DUSP6KO_RG1-14    MIDTLRPGPSRRKWRSARRWRGSTSSWSWATSGCC*
                  MIDTLRPVPSRRKWRSARRWRGSTSSWSWATSGCC*

DUSP6_WT          EDRDRFTRRCGTDTVVLYDESSSDWNENTGGESVLGLLLKKLKDEGCRAFYLEGGFSKFQAEFS    144
DUSP6KO_RG4-9     EDRDRFTRRCGTDTVVLYDESSSDWNENTGSGCCSRSSRTRAAGRSTWKVASVSSKPSSPCIARPI*
DUSP6KO_RG4-10    EDRDRFTRRCGTDTVVLYDESSSDWNENTSRCSGCCSRSSRTRAAGRSTWKVASVSSKPSSPCIARPI*
                  EDRDRFTRRCGTDTVVLYDESSSDWNENTGGEVGARAAAQEAQGRGLPGVLPGRWLQ*
```

Figure S2. Sequencing analyses of *DUSP6* KO cells. (A) Histograms of Sanger sequencing of DNA isolated from *DUSP6* KO cells generated using the RG1 and RG4 sgRNAs. (B) Sequences of DNA isolated from *DUSP6* KO cells. (C) Deduced amino acid sequences of DUSP6 protein isolated *DUSP6* KO cells.

Figure S3

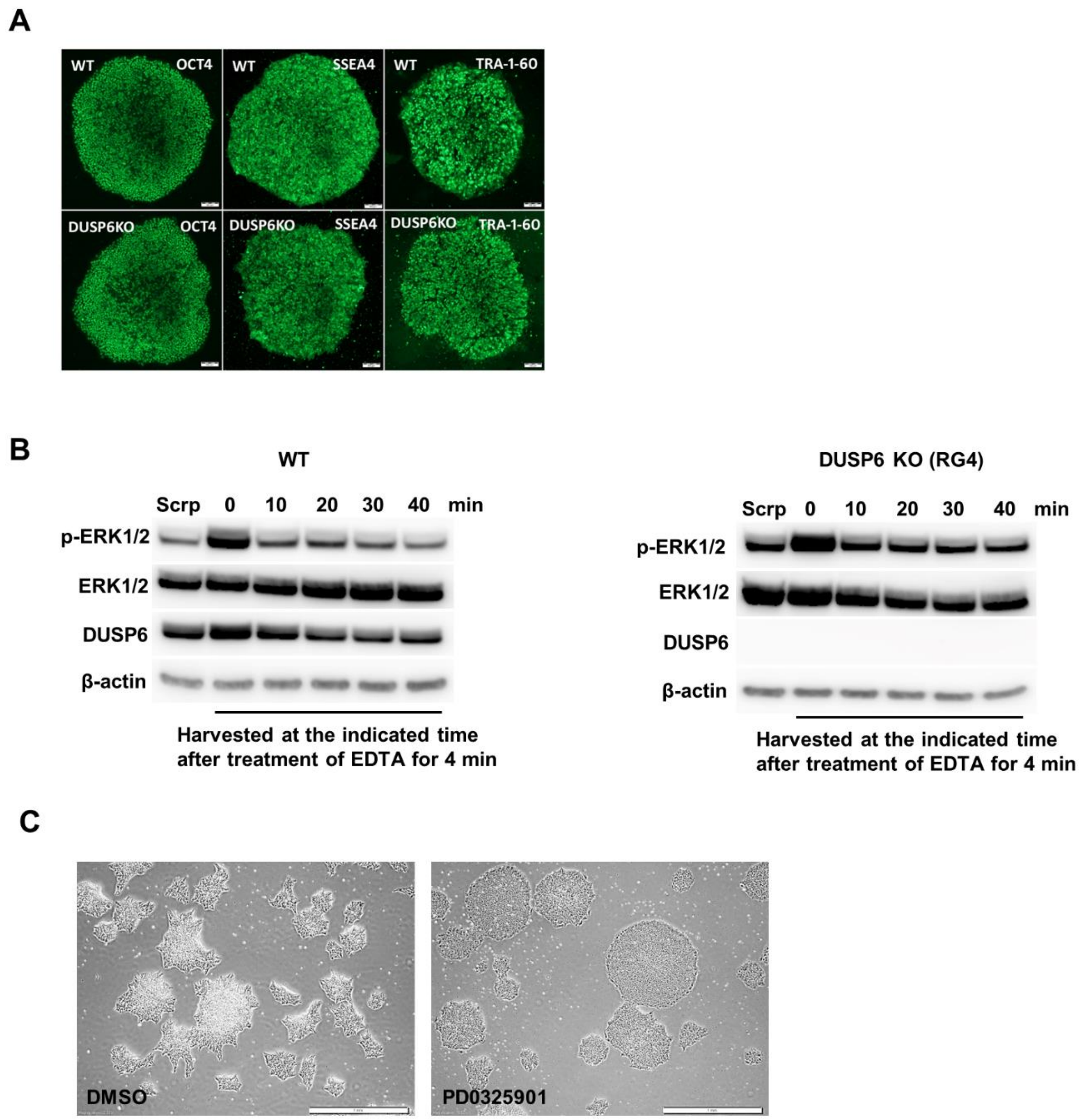

Figure S3. Expression of pluripotency markers and p-ERK in *DUSP6* KO cells. (A) Expression of pluripotency markers, OCT4, SSEA4, and TRA-1-60, in WT and *DUSP6* KO cells. Colonies of WT and *DUSP6* KO cells were stained with the indicated primary antibody and Alexa-480 conjugated secondary antibody (scale bar, 200 μm). (B) Duration of ERK activation following EDTA treatment in WT and *DUSP6* KO cells. (C) Suppression of outgrowth phenotype in KO-A cells by 1 μM PD0325901 treatment for 24 h (scale bar, 1 mm).

Figure S4

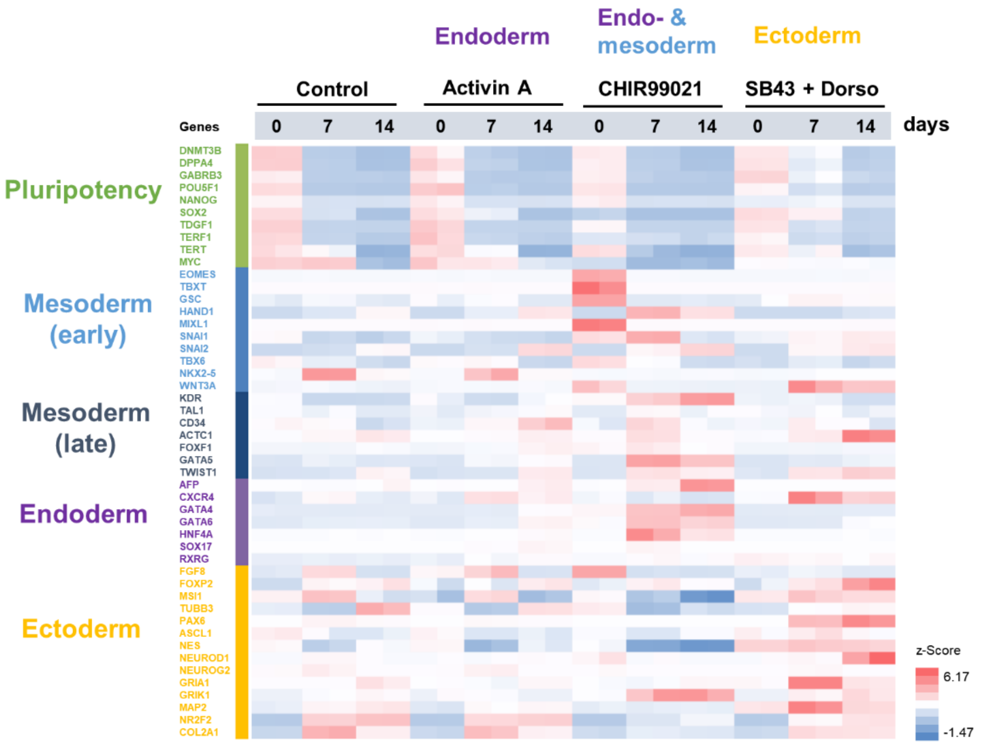

Figure S4. Validation of spontaneous differentiation with 5% FBS in DMEM. As positive controls towards endoderm, mesoderm and ectoderm lineages, WT (hFSiPS1) cells were treated with activin A (50 ng/ml, 48 h) for endoderm or CHIR99021 (8 µM, 24 h) for endo- and mesoderm or SB431542 + dorsomorphin (3µM each, 48 h) for ectoderm before initiating differentiation. After treatment of agents, cells were differentiated by changing media with DMEM containing 5% FBS. Cells were harvested day 0 before differentiation, day 7 and 14 after differentiation. DMSO-treated cells were used as Control.

Figure S5

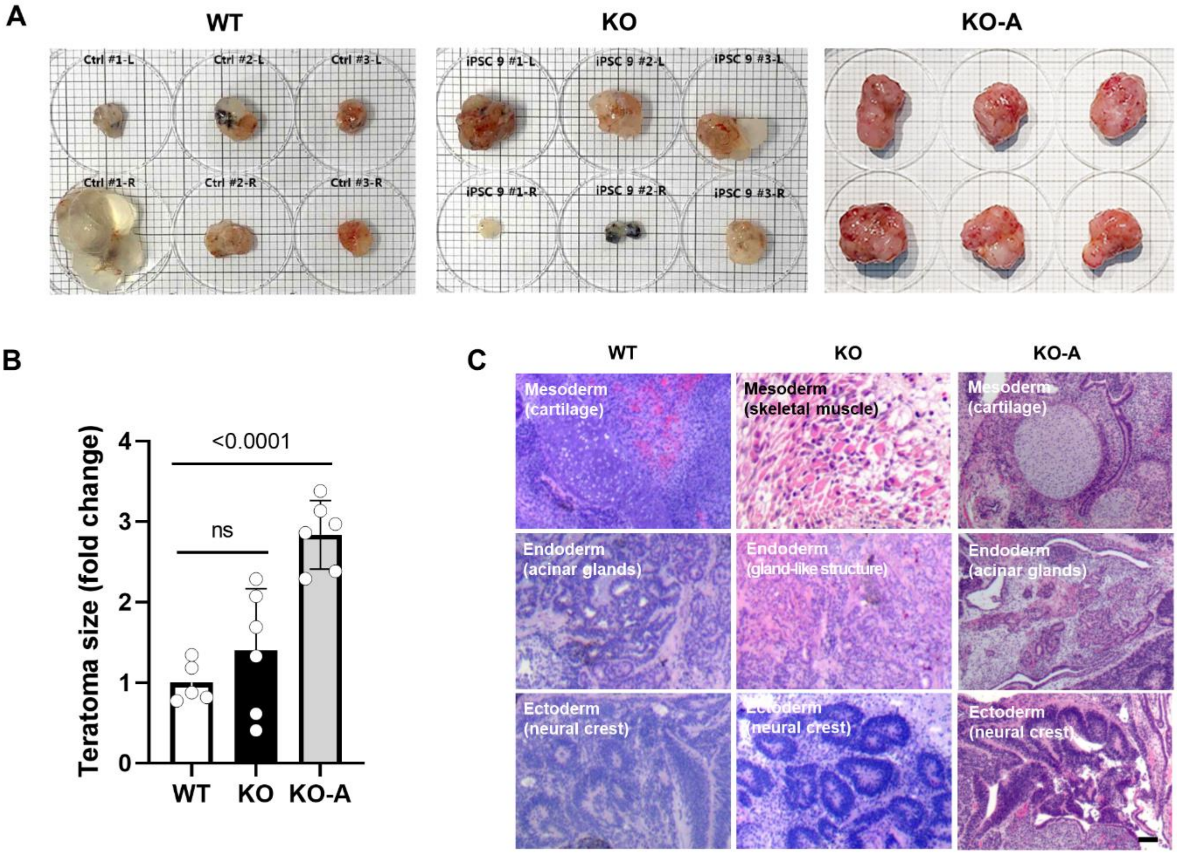

Figure S5. Teratoma assay of WT and DUSP6 knockout (KO) cells. Immune deficient NOD/SCID mice were subcutaneously injected with cell suspensions (1 x 10^6 cells in 100 μL). Teratoma formation was monitored visually for eight weeks. After eight weeks, the mice were euthanasia by CO2 asphyxiation and teratomas were carefully excised from the surrounding tissue. (A) Teratoma excised from mice injected with WT, DUSP6 KO, and DUSP6 KO-A cells, respectively. (B) Comparison of teratoma size. (C) Histochemical analyses to confirm the presence of tissues originated from three germ layers.

Figure S6

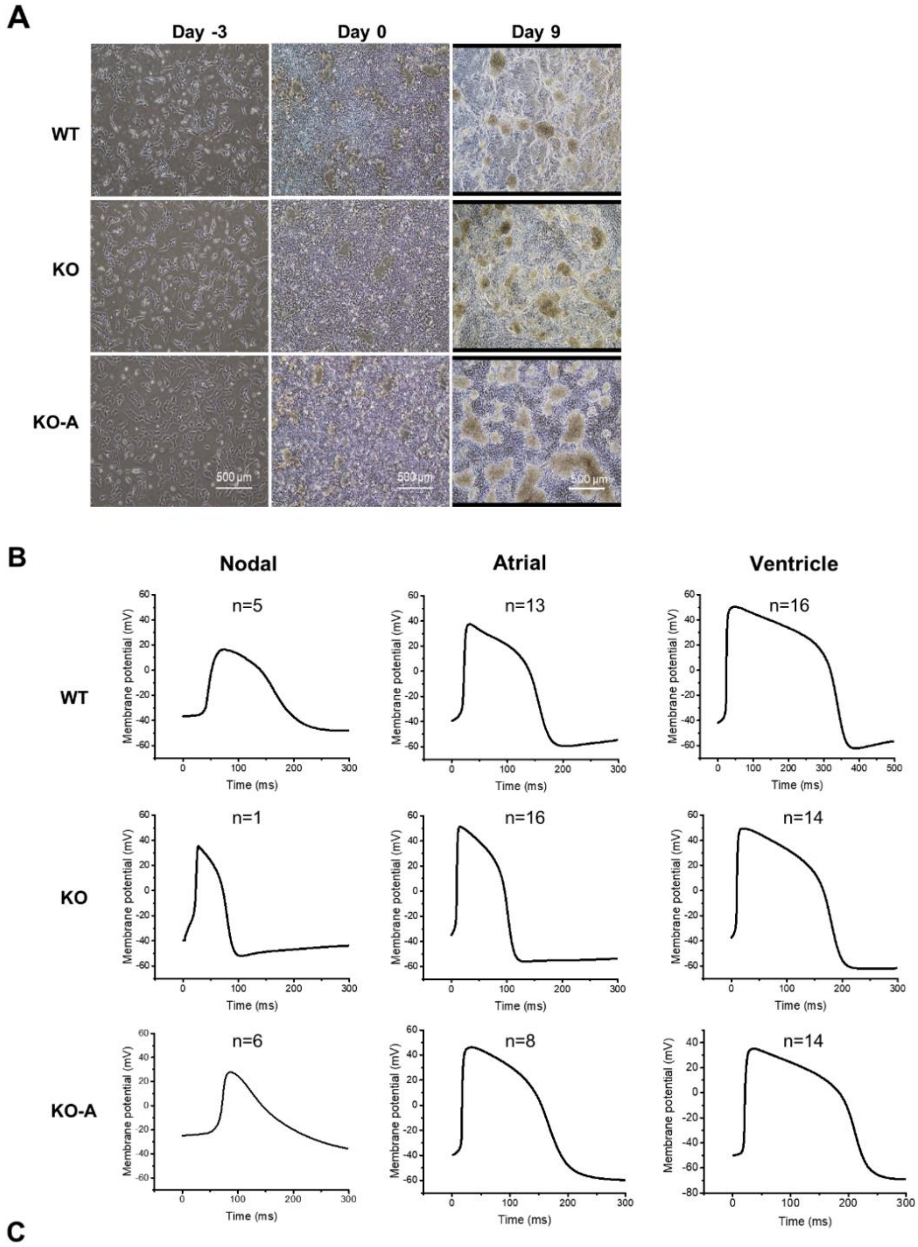

C

Types	Nodal	Atrial	Ventricle
Amplitude	< 100 mV	> 100mV	> 100 mV
V_{max}	< 10 V/s	> 10 V/s	> 10 V/s
APD_{80}	-	< 200 ms	> 200 ms
APD ratio (APD20-30)/(APD70-80)	-	< 2.5	> 2.5

Figure S6. Types of cardiomyocytes derived from WT and *DUSP6* KO cells. (A) Morphology of cells and cardiomyocytes derived from WT, KO, and KO-A cells. (B) Types of cardiomyocytes according to the beating pattern. (C) Categorization indexes of cardiomyocytes.

Western blot data (full size)

Figure 1_F

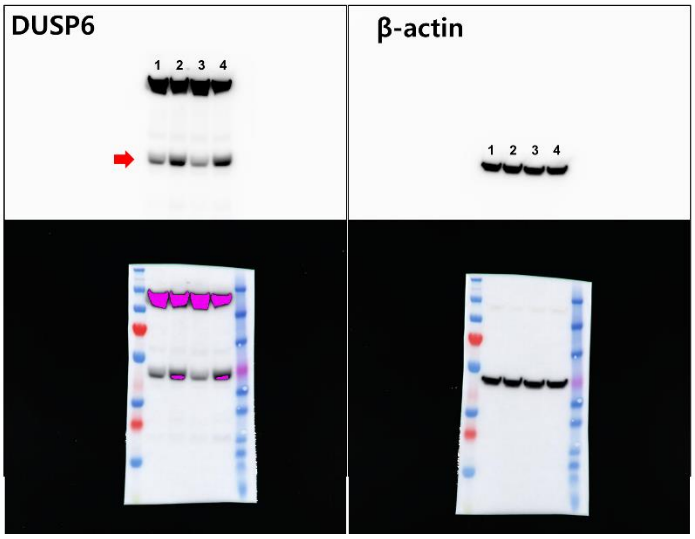

1: Mech (hFSiPS1)
2: EDTA (hFSiPS1)
3: Mech (SNUhES31)
4: EDTA (SNUhES31)

Figure 2_A

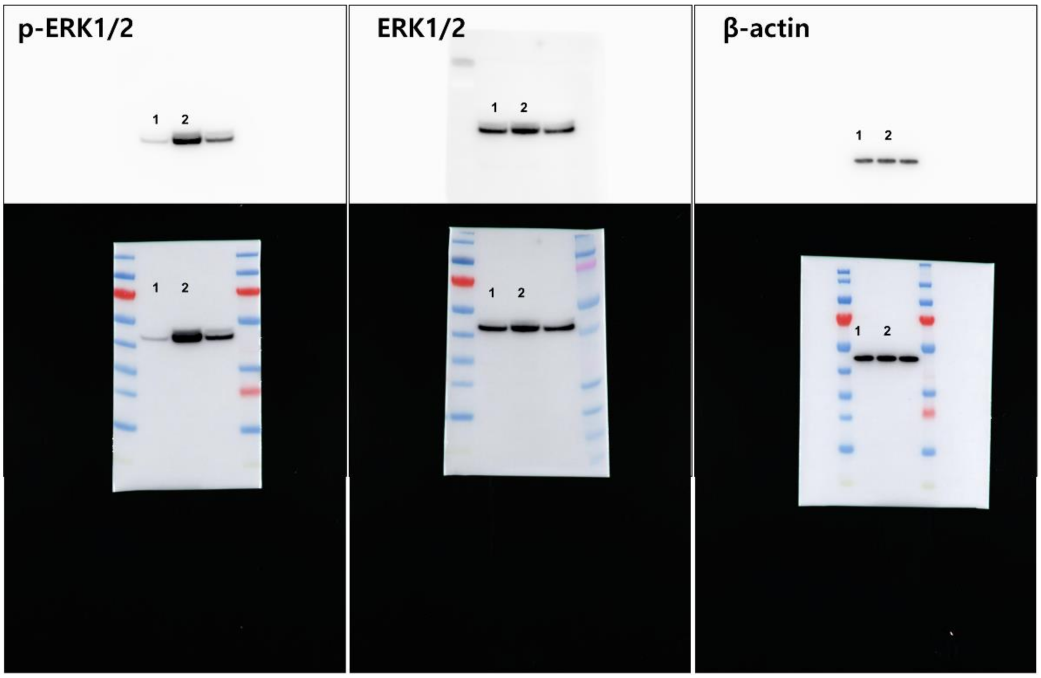

1: Scrp (hFSiPS1)
2: EDTA (hFSiPS1)

Figure 2_C

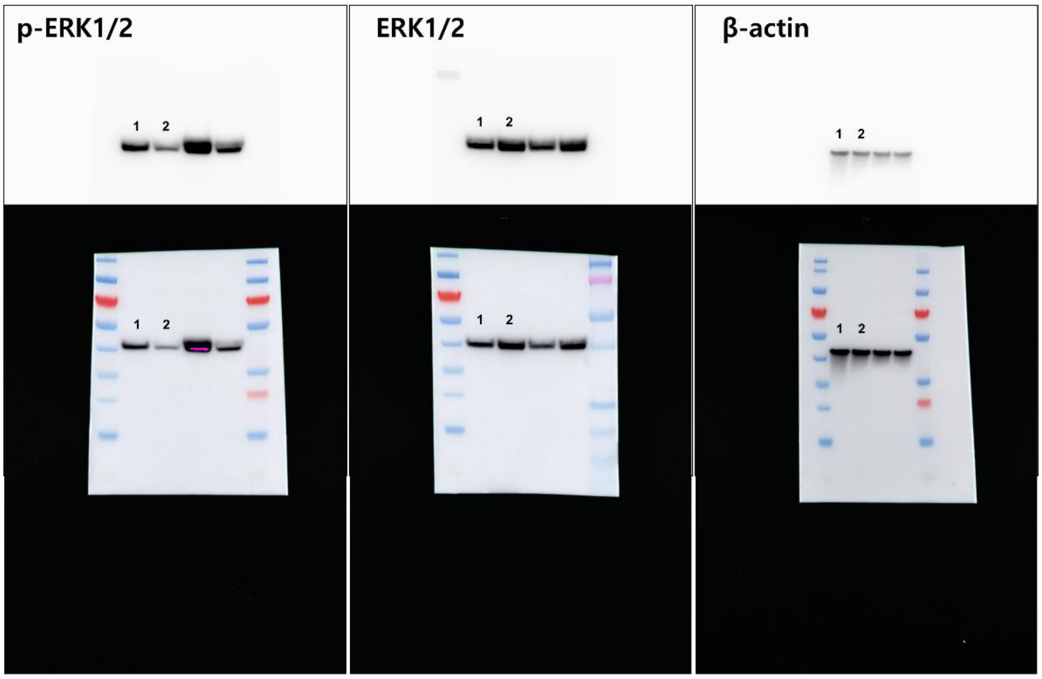

1: Scrp (hFSiPS1)
2: Direct (hFSiPS1)

Figure 2_D

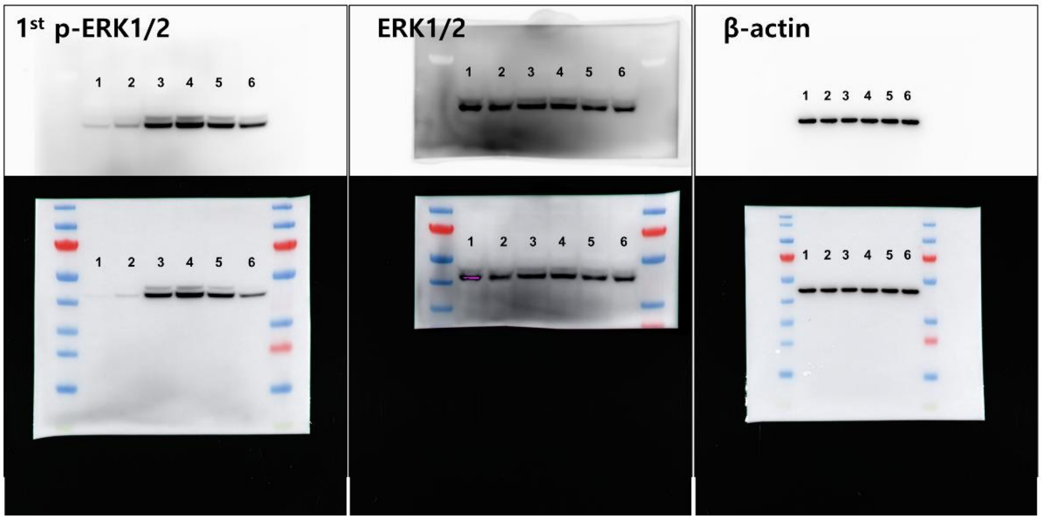

1: Direct
2: Scrp
3: EDTA
4: ReLeSR
5: TrypLE
6: Dispase

Figure 2_D

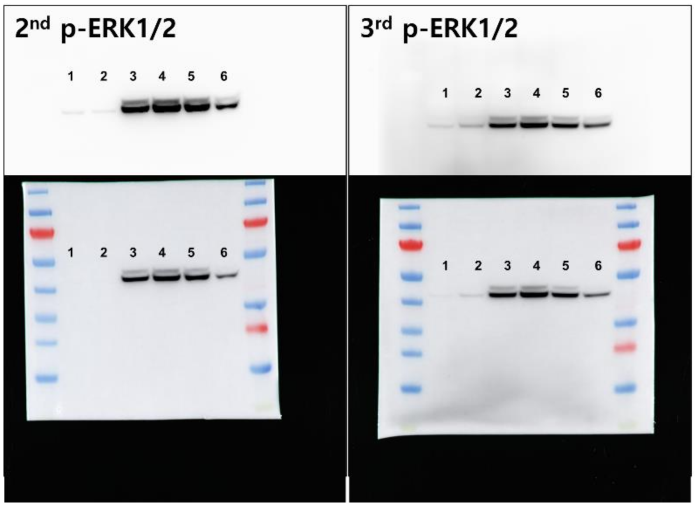

1: Direct
2: Scrp
3: EDTA
4: ReLeSR
5: TrypLE
6: Dispase

Figure 2_G-1

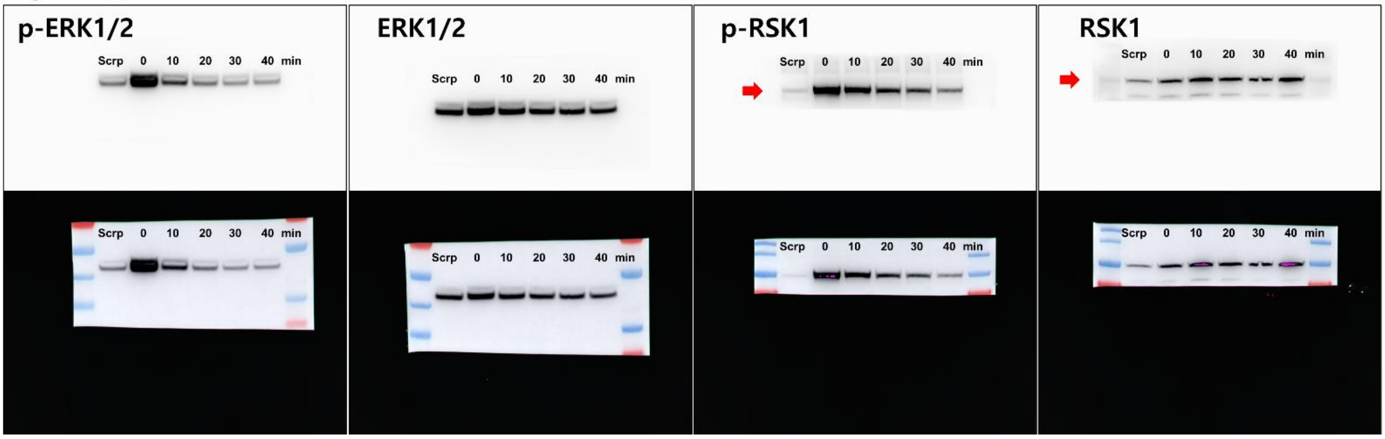

Figure 2_G-2

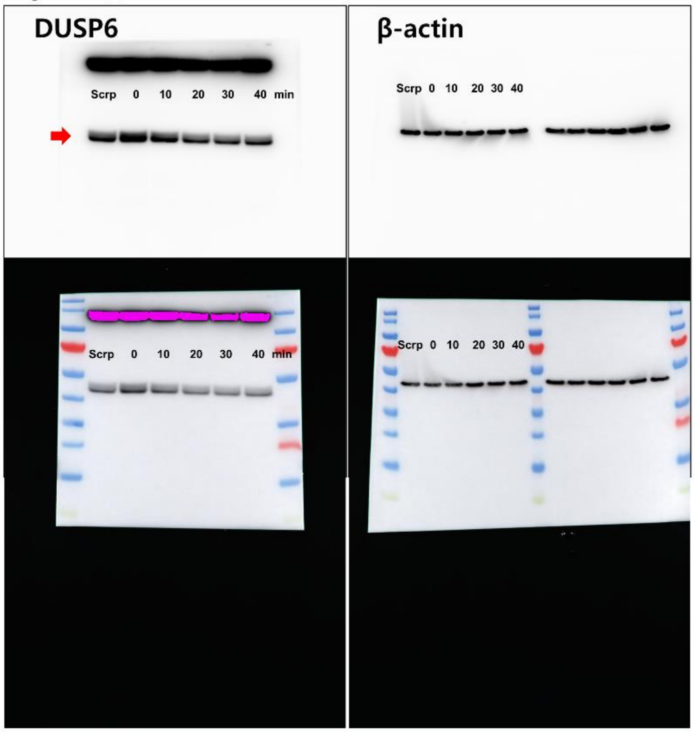

Figure 2_H-1

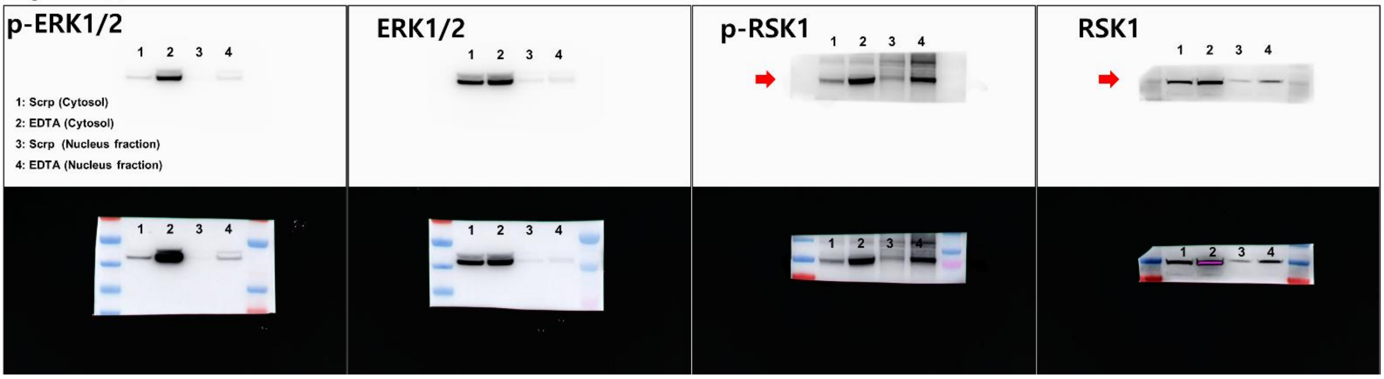

Figure 2_H-2

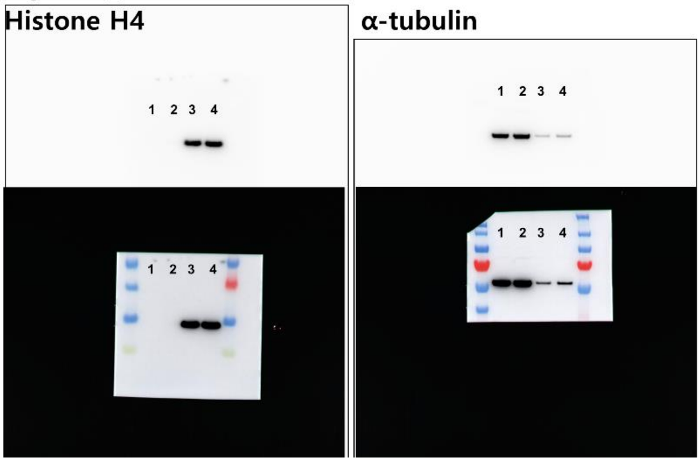

1: Scrp (Cytosol)
2: EDTA (Cytosol)
3: Scrp (Nucleus)
4: EDTA (Nucleus)

Figure 2_K

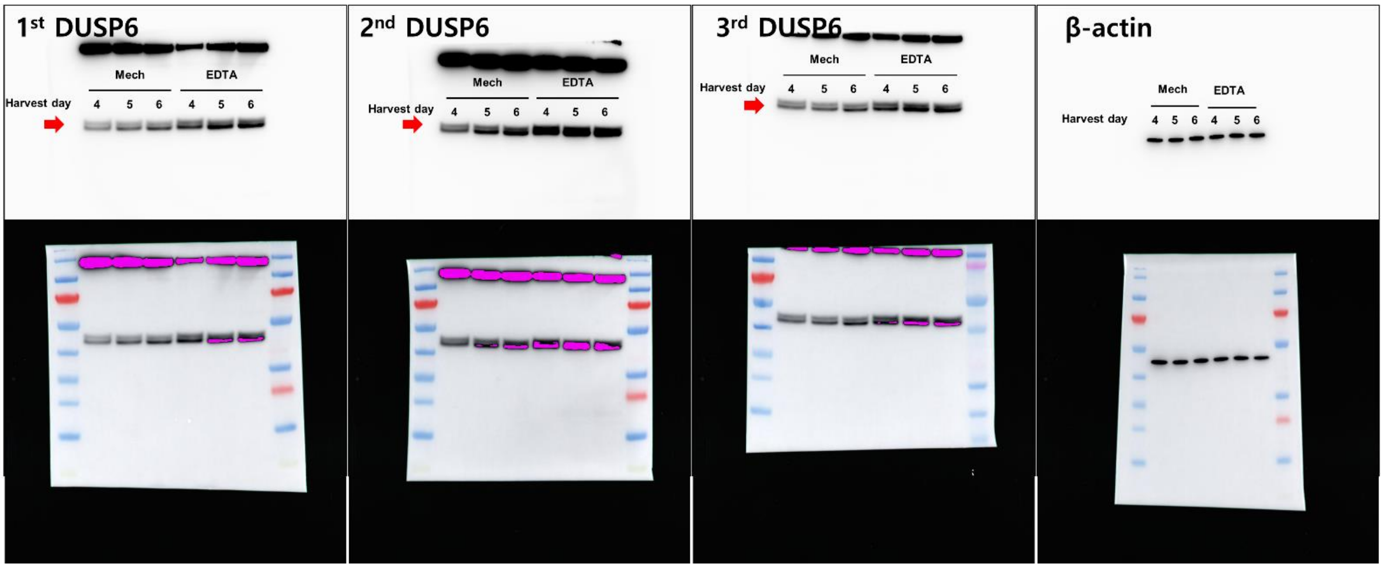

Figure 3_A

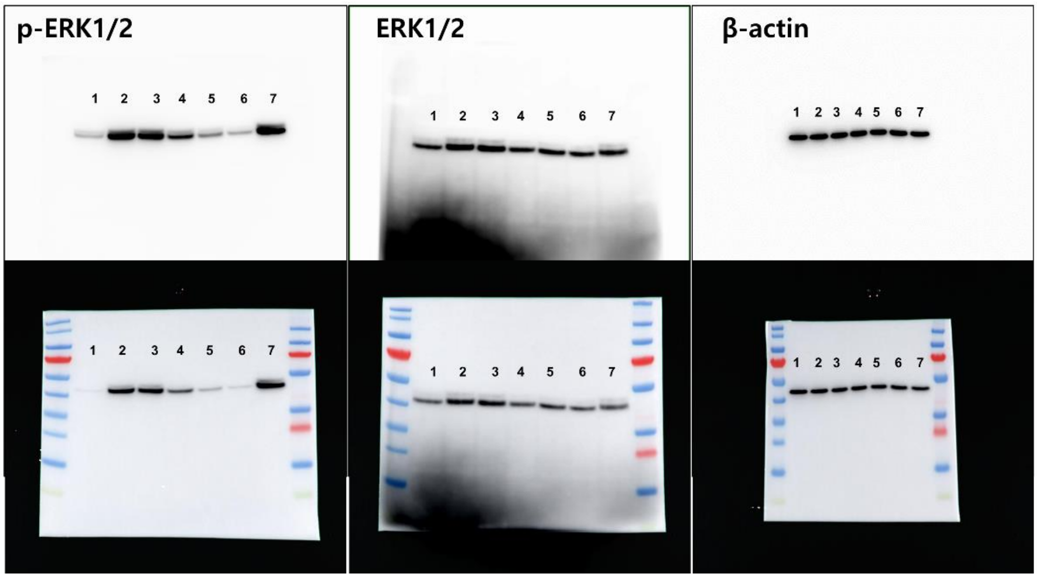

1: Control
2: None (EDTA-treated)
3: PD173074 (EDTA-treated)
4: Y27632 (EDTA-treated)
5: PF573228 (EDTA-treated)
6: Y27+PF57 (EDTA-treated)
7: DMSO (EDTA-treated)

Figure 3_B

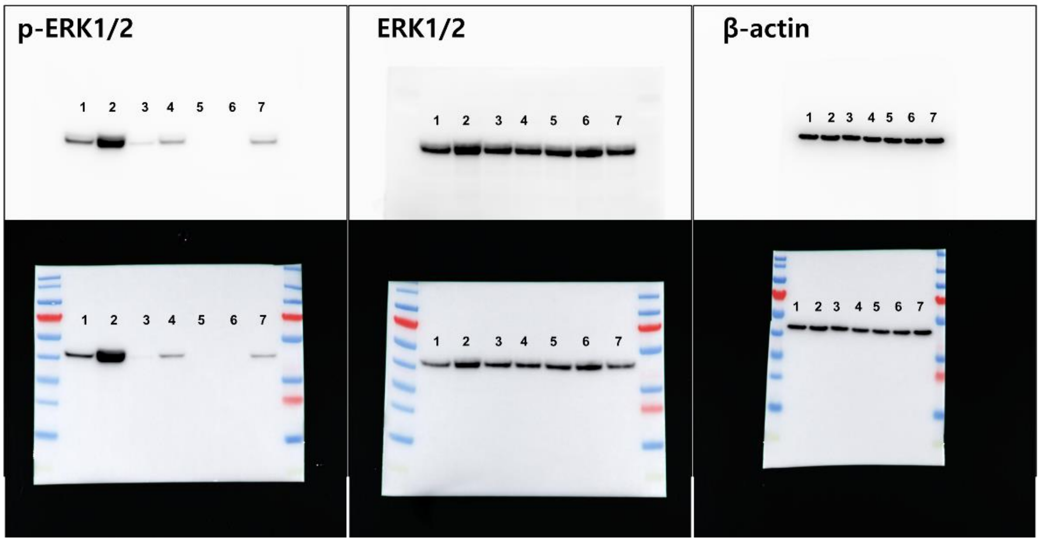

1: Control
2: None (EDTA-treated)
3: PD173074
4: Y27632
5: PF573228
6: Y27+PF57
7: DMSO

Figure 3_C

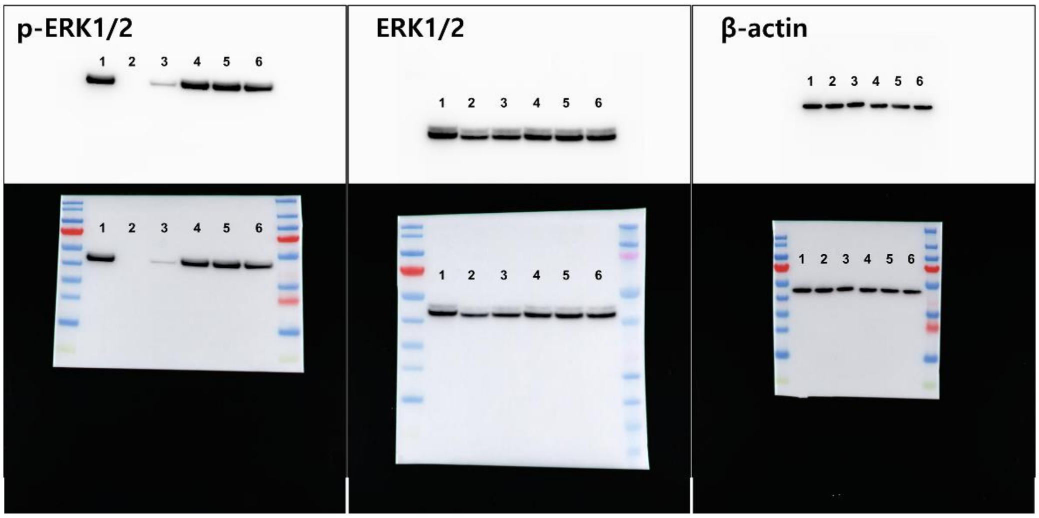

1: None
2: PD0325901 (0.1 μM) treated
3: PF573228 (10 μM) treated
4: PF573228 (1 μM) treated
5: PF573228 (0.1 μM) treated
6: PF573228 (0.01 μM) treated

Figure 4_B

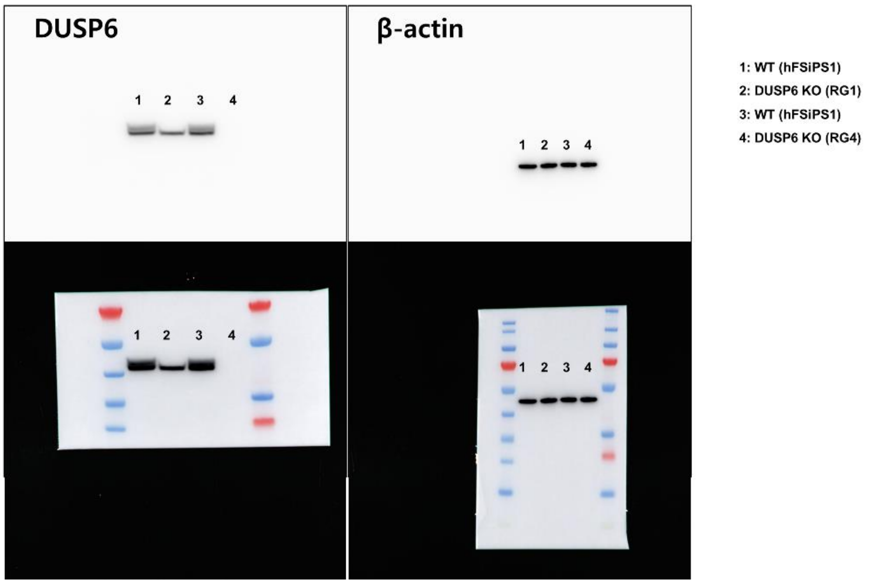

Figure 4_C

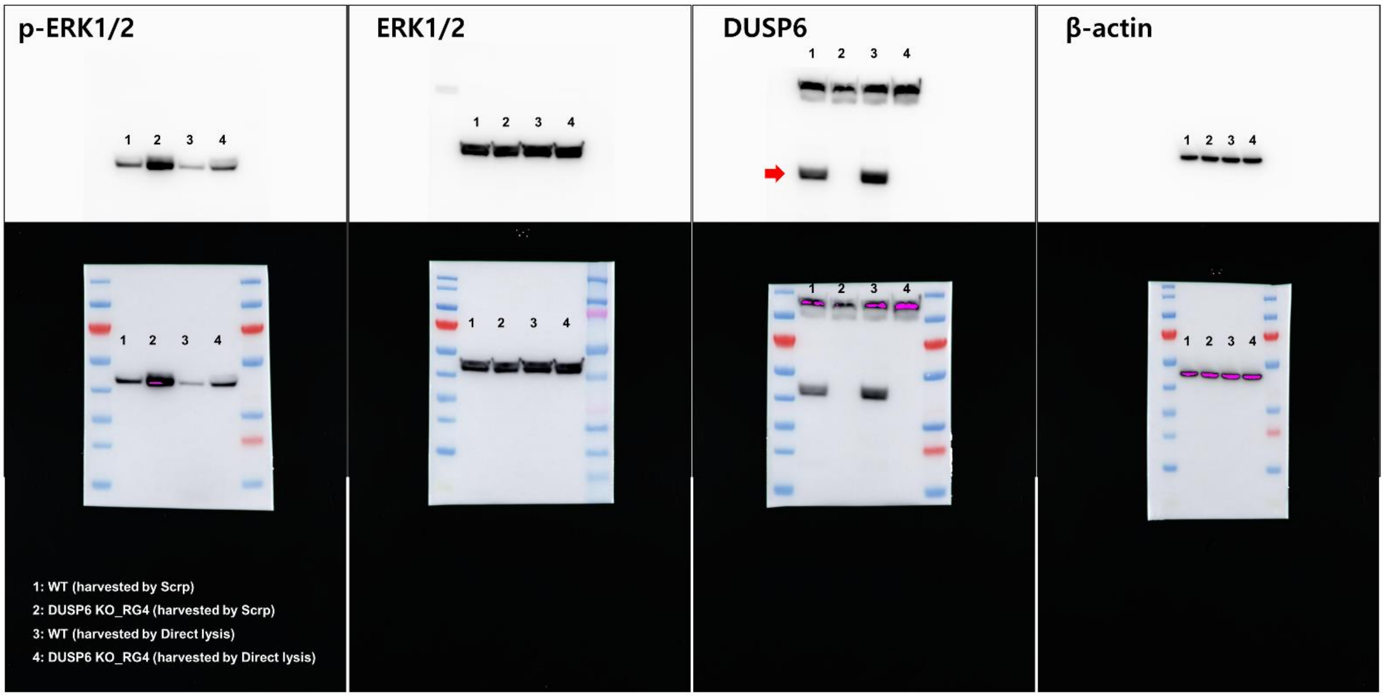

Figure 4_D

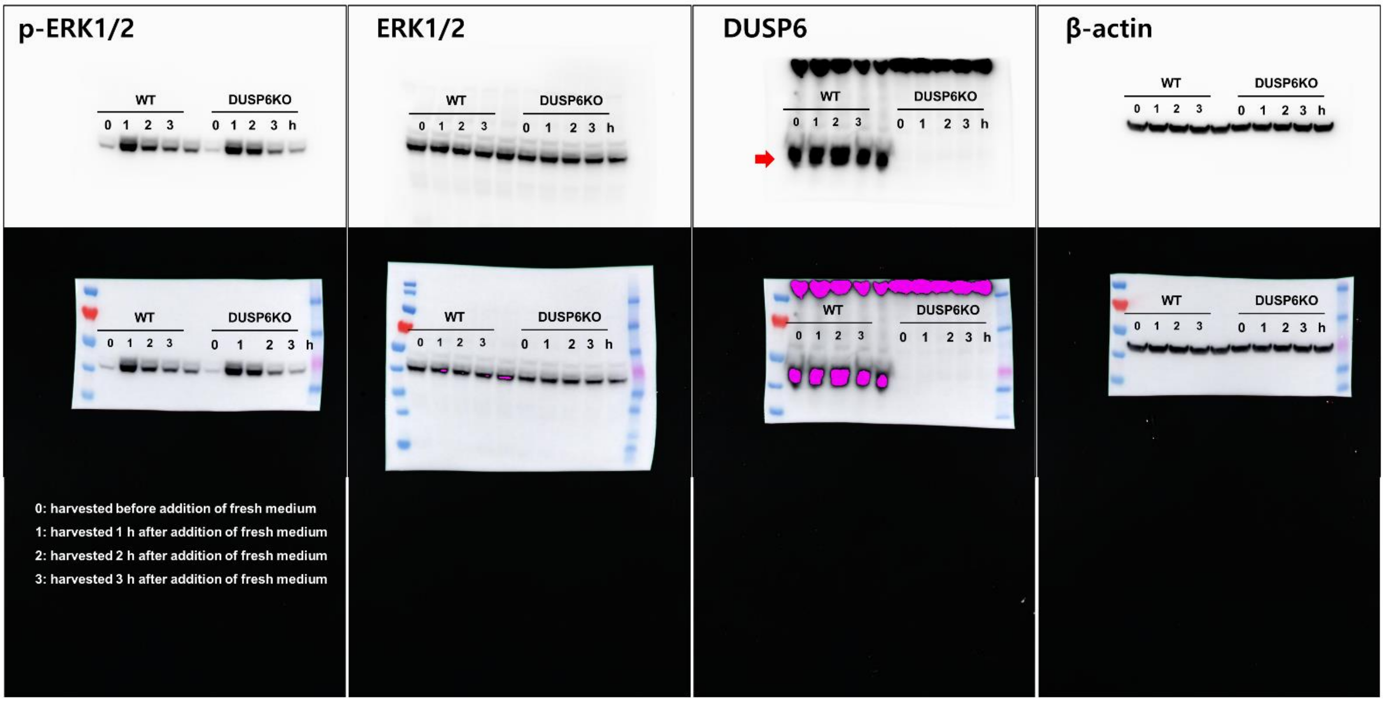

Figure 4_E

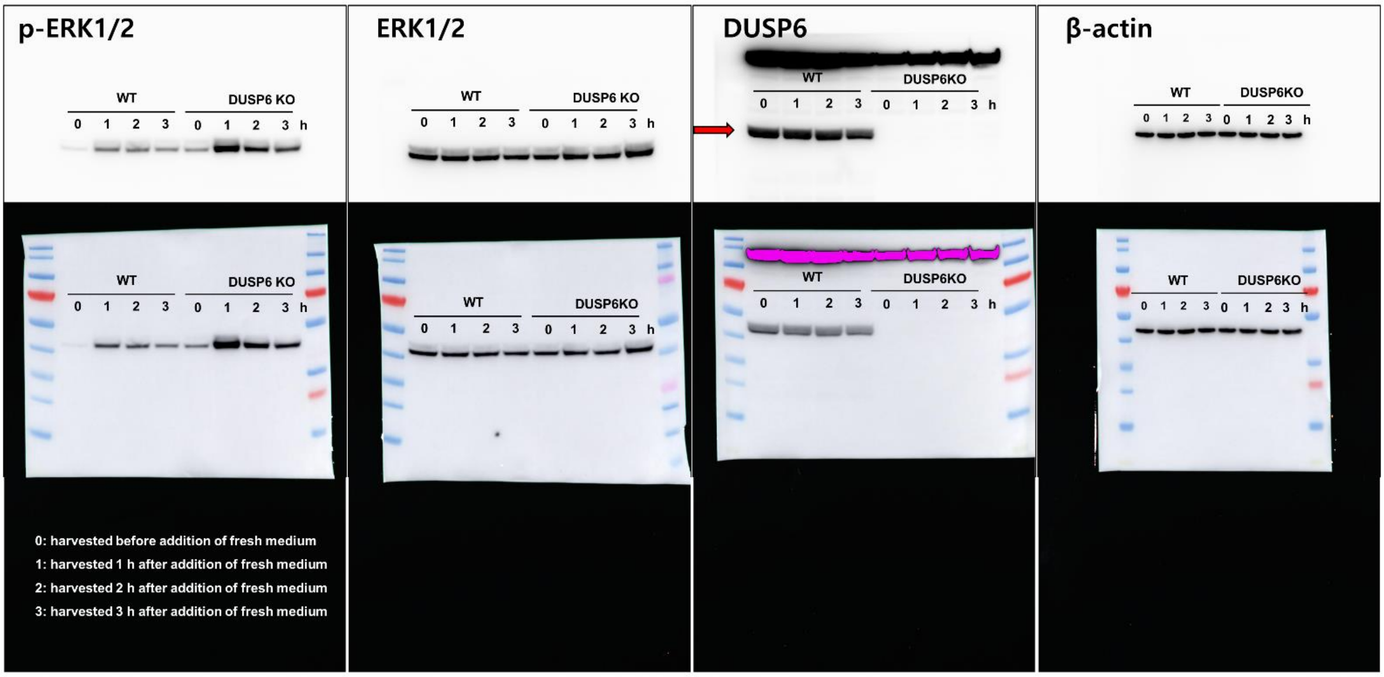

Figure 4_H

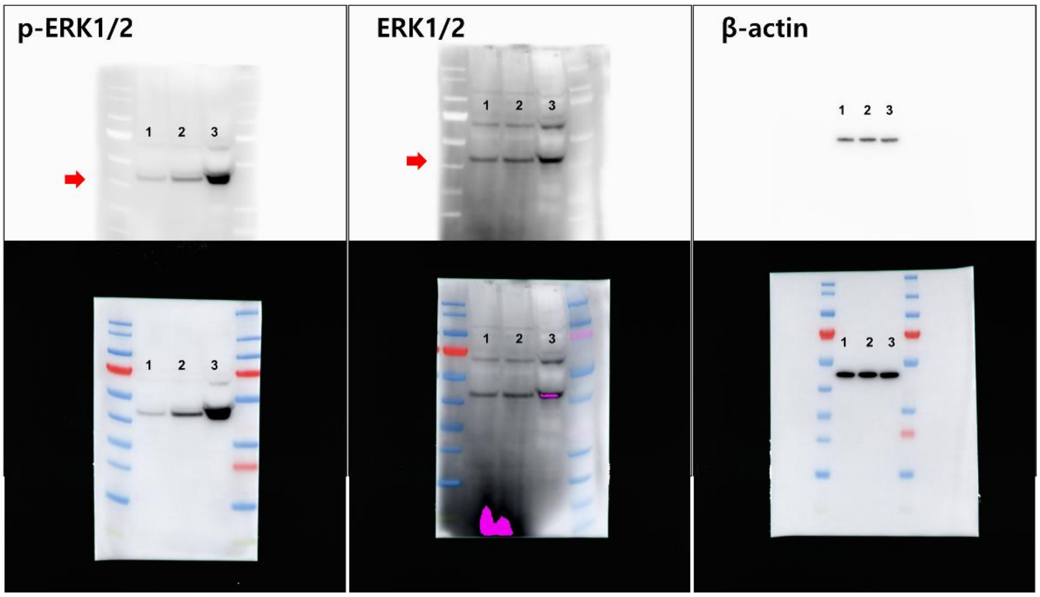

1: WT (hFSiPS1)
2: DUSP6 KO
3: DUSP6 KO-A

Supplementary Figure 3_B
- WT

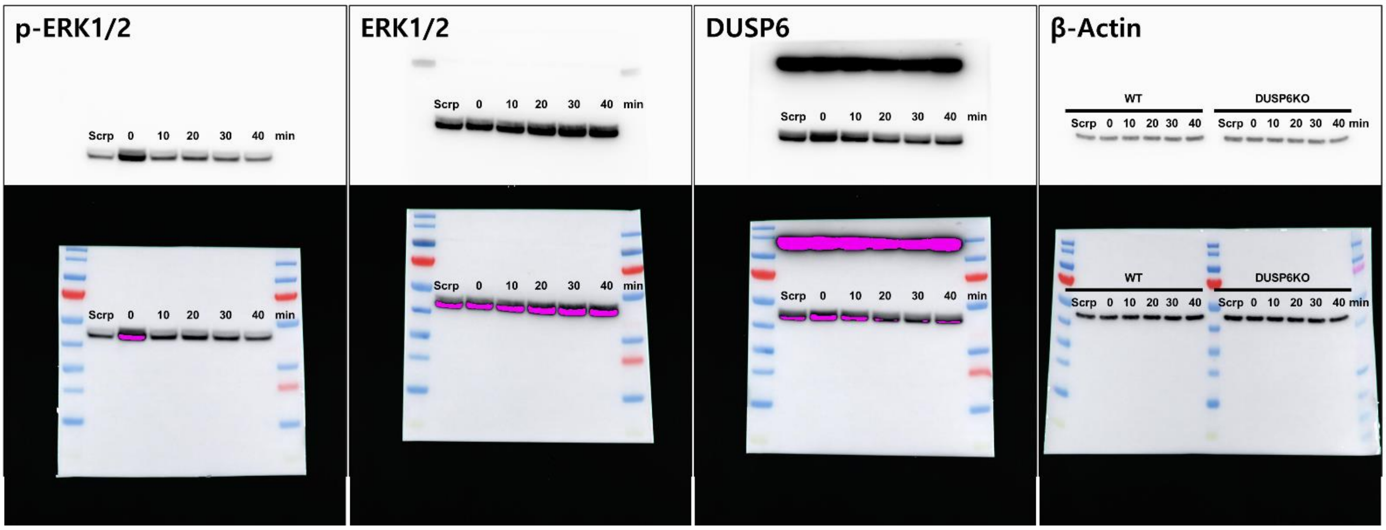

Supplementary Figure 3B
- DUSP6KO

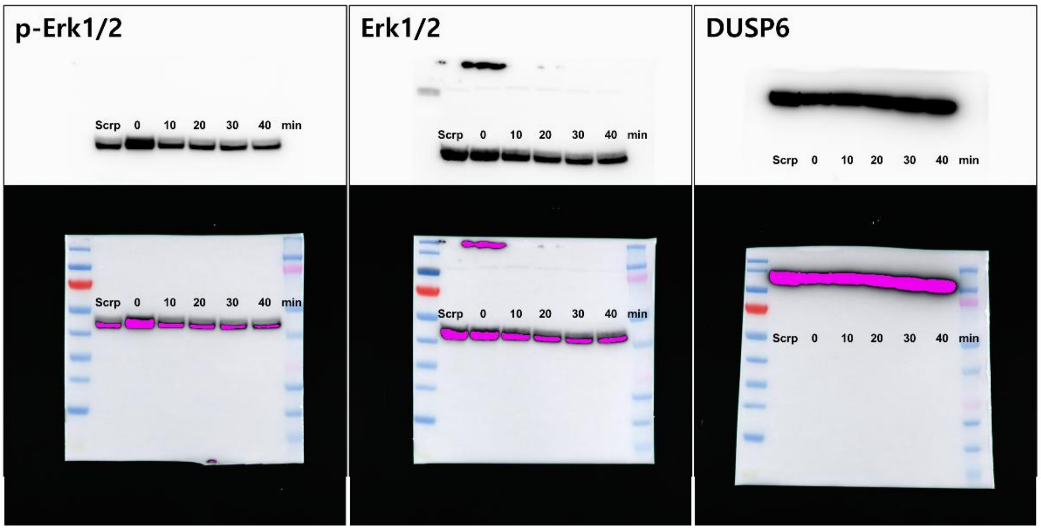

KEY RESOURCE TABLE

REAGENT OR RESOURCE	SOURCE	IDENTIFIER
Antibodies		
Anti-α-Tubulin	Sigma Aldrich	SIG-T6074
Anti-Histone H4 (mono methyl K20)	Abcam	Ab177188
Anti-β-Actin	Sigma Aldrich	A5316
Anti-OCT-3/4 (C-10)	Santa Cruz	Sc-5279
Anti-SSEA-4, clone MC-813-70	Merck Millipore	MAB4304
Anti-TRA-1-60, clone TRA-1-60	Merck Millipore	MAB4360
Anti-DUSP6	Abcam	Ab220811
Anti-Phospho-p44/42 MAPK (ERK1/2) (Thr202/Tyr204) (197G2)	Cell Signaling	#4377S
Anti-p44/42 MAPK (ERK1/2) (L34F12)	Cell Signaling	#4696S
Anti-RSK1 p90 (phospho T573)	Abcam	Ab62324
Anti-RSK1 p90	Abcam	Ab32114
Anti-cTnT	Abcam	Ab209813
Anti-Sarcomeric-α-actinin	Abcam	Ab68167
Anti-MF20	Thermo Fisher	14-6503-82
Donkey anti-Rabbit IgG (H+L) Highly Cross-Adsorbed Secondary Antibody, Alexa Fluor 488	Thermo Fisher	A-21206
Donkey anti-Rabbit IgG (H+L) Highly Cross-Adsorbed Secondary Antibody, Alexa Fluor 594	Thermo Fisher	A-21207
Donkey anti-Mouse IgG (H+L) Highly Cross-Adsorbed Secondary Antibody, Alexa Fluor 488	Thermo Fisher	A-21202
Donkey anti-Mouse IgG (H+L) Highly Cross-Adsorbed Secondary Antibody, Alexa Fluor 594	Thermo Fisher	A-21203
Donkey anti-Rabbit IgG (H+L) Highly Cross-Adsorbed Secondary Antibody, HRP	Thermo Fisher	A16035
Donkey anti-Mouse IgG (H+L) Highly Cross-Adsorbed Secondary Antibody, HRP	Thermo Fisher	A16017
Chemicals		
CCK-8 kit	Dojindo	CK04-20
PD0325901	Sigma Aldrich	PZ0162
PD173074	Sigma Aldrich	P2499

Y27632 dihydrochloride	Sigma Aldrich	Y0503
PF573228	Selleckchem	S2013
CHIR-99021	Selleckchem	CT99021
Wnt-C59	Selleckchem	S7037
DMSO	VWR Life Science	97063-136
DAPI Solution	Thermo Fisher	62248
DyLight™ 594 Phalloidin	Cell Signaling	12877S
RIPA Lysis and Extraction Buffer	Thermo Fisher	8990
Halt™ Protease and Phosphatase Inhibitor Single-Use Cocktail (100X)	Thermo Fisher	78442
Bolt® 4-12% Bis-Tris Plus Gels, 1.0 mm, 15-well	Invitrogen	NW04125
Bolt® 4-12% Bis-Tris Plus Gels, 1.0 mm, 10-well	Invitrogen	NW04120
iBind™ Flex Western Starter Kit	Thermo Fisher	SLF2000S
20X Bolt™ MES SDS Running Buffer	Thermo Fisher	B0002
4% Paraformaldehyde Phosphate Buffer Solution	Fujifilm Wako Pure Chemical	163-20145
Nunc™ Lab-Tek™ II CC2™ Chamber Slide System (2 wells)	Nunc	154852PK
Bovine Serum Albumin solution	Sigma Aldrich	A8412
DPBS (without Ca2+, Mg2+)	Gibco	14190-144
TWEEN 20	LPS	TW2001
Plasmid Midi Kit	Qiagen	12145
Recombinant Cas9 protein	Toolgen	TGEN_CP1
TrypLE	Gibco	12563-011
Accutase	Stem Cell Technologies	05872
Dispase	Stem Cell Technologies	07923
**Critical commercial assays**		
Maxwell® RSC simply RNA Cells Kit	Promega	AS1390
RNA to cDNA EcoDry Premix (Oligo dT)	Clontech	639543
TaqMan Gene Expression assays DUSP6 (Hs04329643_s1)	Thermo Fisher	4331182
TaqMan Gene Expression assays NANOG (Hs0287400_g1)	Thermo Fisher	4331182
TaqMan Gene Expression assays POU5F1 (Hs00999632_g1)	Thermo Fisher	4331182
TaqMan Gene Expression assays SOX2	Thermo Fisher	4331182

(Hs04234836_s1)		
TaqMan Gene Expression assays GAPDH (Hs02786624_g1)	Thermo Fisher	4331182
TaqMan Gene Expression assays ACTB (Hs03023943_g1)	Thermo Fisher	4331182
TaqMan Gene Expression assays MAPK1 (Hs01046830_m1)	Thermo Fisher	4331182
TaqMan Gene Expression assays MYH7 (Hs01110632_m1)	Thermo Fisher	4331182
TaqMan Gene Expression master mix	Thermo Fisher	4369016
TaqMan® hPSC Scrorecard™ Panel 2 X 96w FAST	Thermo Fisher	A15876
Experimental models: cell lines		
hFSiPS1	Korea National Stem Cell Bank	
SNUhES31	Seoul National University	
Oligonucleotides		
sgRNA for DUSP6 (RG1) ATTTCCGACGCGAAGGGCACGGG	Toolgen	
sgRNA for DUSP6 (RG2) GATCGCCATTTCCGACGCGAAGG	Toolgen	
sgRNA for DUSP6 (RG3) AGCGCCGGGTGAAGCGGTCCCGG	Toolgen	
sgRNA for DUSP6 (RG4) CGAGAATACGGGCGGCGAGTCGG	Toolgen	
PCR and sequencing primers for DUSP6KO screening		
RG1_F1 GATTTGAGGTGCAGCCTTGG	Bioneer	
RG1_R1 GGCGCGTACCTTCCAGGTAG	Bioneer	
RG1_SF1 CAACCGCTAGCCTCGG	Bioneer	
RG1_SR1	Bioneer	

TGCGACGACTCGTATAGCTC		
RG4_F1<br><br>GGCATCATGCTGCGGCGCCT	Bioneer	
RG4_R1<br><br>CTGGTGCGGAACGCGCGGTT	Bioneer	
RG4_SF1<br><br>GAGGACCGGGACCGCTTCAC	Bioneer	
RG4_SR1<br><br>GGAGTTCCCTGGGCGCGTAC	Bioneer	
Software and algorithms		
GraphPad Prism 9 version 9.5.1	GraphPad	
Excel 2016	Microsoft	
ImageJ 1.52a	NIH	

DEGs list (ordered in with high average RPKM values first, MAPK signaling pathway-related genes are marked bold)

Gene_ID	Gene_Symbol	Average (RPKM)
**1848**	**DUSP6**	**36.657**
1277	COL1A1	33.438
2014	EMP3	20.117
6781	STC1	14.674
3949	LDLR	12.553
9582	APOBEC3B	11.230
6607	SMN2	10.169
1958	EGR1	10.006
57687	VAT1L	9.999
10893	MMP24	9.313
283576	ZDHHC22	8.656
50487	PLA2G3	8.594
90987	ZNF251	8.304
1592	CYP26A1	8.214
7425	VGF	7.404
**2261**	**FGFR3**	**7.350**
153572	IRX2	7.214
266917	D21S2088E	7.056
8061	FOSL1	6.241
10637	LEFTY1	6.121
10345	TRDN	6.088
388	RHOB	5.742
347694	ECEL1P2	5.434
84152	PPP1R1B	5.037
**3725**	**JUN**	**4.935**
25759	SHC2	4.766
**7010**	**TEK**	**4.734**
4636	MYL5	4.019
7262	PHLDA2	3.860
25840	METTL7A	3.417
4838	NODAL	3.378
6505	SLC1A1	3.331
7980	TFPI2	3.300
1043	CD52	3.299
**784**	**CACNB3**	**3.232**
55885	LMO3	2.905

50624	CUZD1	2.903
90102	PHLDB2	2.690
59	ACTA2	2.649
3400	ID4	2.575
79026	AHNAK	2.043
84691	FAM71F1	2.038
1674	DES	2.007
7044	LEFTY2	2.005
153571	C5orf38	1.863
91947	ARRDC4	1.759
11045	UPK1A	1.742
8620	NPFF	1.624
56977	STOX2	1.584
3040	HBA2	1.567
401089	FOXL2NB	1.545
56142	PCDHA6	1.351
54757	FAM20A	1.189
100132287	LOC100132287	1.120
341405	ANKRD33	1.019
80031	SEMA6D	1.014
54103	GSAP	0.965
6376	CX3CL1	0.921
5142	PDE4B	0.914
4879	NPPB	0.871
1259	CNGA1	0.780
57593	EBF4	0.761
207107	SFTA1P	0.731
116986	AGAP2	0.637
164284	APCDD1L	0.637
283392	TRHDE-AS1	0.633
105377623	LOC105377623	0.593
**1850**	**DUSP8**	**0.563**
78998	RHPN1-AS1	0.548
10265	IRX5	0.538
10417	SPON2	0.498
339674	LINC00634	0.480
139189	DGKK	0.478
1641	DCX	0.437
9586	CREB5	0.436

8092	ALX1	0.434
30818	KCNIP3	0.427
145447	ABHD12B	0.417
105369758	DDN-AS1	0.414
863	CBFA2T3	0.394
5153	PDE1B	0.390
9890	PLPPR4	0.387
4004	LMO1	0.371
79192	IRX1	0.364
4921	DDR2	0.364
285025	CCDC141	0.354
3754	KCNF1	0.351
26509	MYOF	0.351
378807	CATSPER4	0.349
8788	DLK1	0.344
64211	LHX5	0.334
284353	NKPD1	0.329
3037	HAS2	0.329
148398	SAMD11	0.320
6335	SCN9A	0.293
23462	HEY1	0.285
1960	EGR3	0.284
105374042	LOC105374042	0.280
55504	TNFRSF19	0.276
51176	LEF1	0.275
57053	CHRNA10	0.268
57526	PCDH19	0.240
107984773	LINC01852	0.236
93349	SP140L	0.236
10512	SEMA3C	0.235
400120	SERTM1	0.228
3687	ITGAX	0.225
84645	C22orf23	0.216
645158	CBX3P2	0.210
320	APBA1	0.207
7225	TRPC6	0.201
**284**	**ANGPT1**	**0.192**
27303	RBMS3	0.185
1775	DNASE1L2	0.183

100526740	ATP5J2-PTCD1	0.180
2825	GPR1	0.179
360200	TMPRSS9	0.179
7273	TTN	0.175
2162	F13A1	0.162
5173	PDYN	0.145
**2246**	**FGF1**	**0.143**
3736	KCNA1	0.137
1004	CDH6	0.131
100532735	INO80B-WBP1	0.128
**93589**	**CACNA2D4**	**0.118**
146850	PIK3R6	0.115
115111	SLC26A7	0.112
84674	CARD6	0.108
6506	SLC1A2	0.108
83450	DRC3	0.107
11189	CELF3	0.105
64084	CLSTN2	0.102
8989	TRPA1	0.100
55286	C4orf19	0.096
5630	PRPH	0.091
395	ARHGAP6	0.089
3624	INHBA	0.080
57234	LINC00869	0.074
100529855	ZNF625-ZNF20	0.073
1030	CDKN2B	0.067
80000	GREB1L	0.058
57282	SLC4A10	0.053
2346	FOLH1	0.048
401093	MBNL1-AS1	0.033
